# The anti-tumour activity of TNF in melanoma is determined by cFLIP

**DOI:** 10.1038/s41419-026-09154-6

**Published:** 2026-08-01

**Authors:** Johanna Stachelscheid, Cäcilia Kaul, Katrin Gerstenberg, J. Paul Werthenbach, Fabian Schorn, Joy Steinkamp, Paola Zigrino, Manolis Pasparakis, Lars M. Schiffmann, Hamid Kashkar

**Affiliations:** 1 University of Cologne, Faculty of Medicine and University Hospital of Cologne, Institute for Molecular Immunology, Cologne, Germany; 2 Department I of Internal Medicine, University of Cologne, Faculty of Medicine and University Hospital of Cologne, Cologne, Germany; 3 Department of Dermatology and Venereology, University of Cologne, Faculty of Medicine and University Hospital of Cologne, Cologne, Germany; 4 University of Cologne, Faculty of Mathematics and Natural Sciences, Institute for Genetics, Cologne, Germany; 5 University of Cologne, Faculty of Medicine and University Hospital of Cologne, Center for Molecular Medicine Cologne (CMMC), Cologne, Germany; 6 University of Cologne, Faculty of Medicine and University Hospital of Cologne, Cluster of Excellence Cellular Stress Responses in Aging-associated Diseases (CECAD), Cologne, Germany; 7 University of Cologne, Faculty of Medicine and University Hospital of Cologne, Department of General, Visceral, Thoracic and Transplant Surgery, Cologne, Germany

**Keywords:** Cell death, Melanoma

## Abstract

Tumour necrosis factor (TNF) is a pleiotropic cytokine originally identified for its ability to kill cancer cells. However, a paradoxical tumour-promoting role for TNF emerged when early attempts to exploit its anti-tumour activity in cancer therapy produced conflicting outcomes, raising the question of whether TNF should be viewed as a therapeutic agent or a treatment target in cancer. Here, we demonstrate that expression of cFLIP, a catalytically inactive paralogue of caspase-8 (CASP8), determines the susceptibility of melanoma cells to TNF and thereby controls melanoma growth in a syngeneic, immune-competent mouse model of B16F10 cutaneous melanoma. B16F10 melanoma cells lacking cFLIP (*cFlip*^*KO/KO*^ cells) failed to grow in wild-type mice, whereas in TNF-deficient mice, *cFlip*^*KO/KO*^ melanoma cells formed palpable tumours and exhibited robust subcutaneous growth. These findings indicate that TNF alone is sufficient to control melanoma growth in the absence of cFLIP. Importantly, the anti-tumour activity of TNF has predominantly been investigated through targeting cellular inhibitors of apoptosis proteins (cIAPs), which promotes RIPK1 activation and TNF-induced cytotoxicity. We show that genomic ablation of cIAPs or RIPK1, in contrast to cFLIP, neither triggered TNF-induced toxicity nor affected melanoma growth in vivo. Collectively, our data underscore the central role of cFLIP in regulating melanoma responses to TNF and suggest that endogenous immune surveillance as well as immunotherapies involving TNF could strongly benefit from cFLIP targeting strategies.

## Introduction

Cutaneous melanoma is a devastatingly aggressive malignancy arising through the malignant transformation of melanocytes. During this process, melanoma cells acquire the capability to evade immune surveillance and resist diverse cytotoxic insults [1]. Dysregulation of cell death is an important hallmark of melanoma, with extensive genetic, functional, and biochemical evidence identifying apoptosis resistance as a key driver of tumour progression and therapy failure [1, 2]. The mitochondrial apoptotic pathway involving the BCL2 protein family and the initiator caspase-9 (CASP9) mainly determines the chemosensitivity of melanoma. However, therapeutic targeting of mitochondrial apoptosis using the BCL-2 inhibitor venetoclax has shown limited efficacy. The extrinsic apoptotic pathway involving death receptors (DRs) and the initiator caspase-8 (CASP8; also called FLICE) is decisive for the immune surveillance of melanoma and novel cancer immunotherapies (e. g. PD-1/PD-L1) [3]. Therapeutic value of targeting the extrinsic apoptotic pathway in melanoma has not been explored.

Apoptosis initiated by DRs such as FAS or TNF-related apoptosis-inducing ligand (TRAIL) receptors (TRAIL-Rs) relies on the formation of a death-inducing signalling complex (DISC), including the adaptor protein FADD and CASP8 [4]. Activation of TNF receptor-1 (TNF-R1) leads to the association of TNF-R1-associated DD protein (TRADD) and receptor interacting kinase 1 (RIPK1) and formation of complex I. TRADD binds TNF-R associated factor 2 (TRAF2), which further recruits ubiquitin ligases, the cellular inhibitor of apoptosis protein 1 (cIAP1) and cIAP2. cIAPs ubiquitylate RIPK1 and other components of complex I and promote NF-κB and MAPK signalling. TNF-R1 induces apoptosis when cytosolic complex II is nucleated by either TRADD or RIPK1, followed by recruitment of FADD and association and activation of CASP8. The cytosolic complex consisting of TRADD-FADD-CASP8 (complex IIa) can be formed when cells are exposed to cycloheximide (CHX). When RIPK1 ubiquitylation is diminished by blocking/ablating cIAPs (e.g. treatment with birinapant), the cytosolic complex consisting of RIPK1-FADD-CASP8 (complex IIb) can be formed [5, 6]. Under both conditions, CASP8 activates downstream executioner caspases, CASP3 and CASP7, leading to apoptosis.

Beyond apoptosis induction, CASP8 also functions as a critical survival factor and prevents necroptosis by cleaving RIPK1 and RIPK3. Inhibition of CASP8 during TNF signalling unleashes RIPK1 and RIPK3 kinase activity, leading to phosphorylation of pseudokinase mixed lineage kinase domain-like (MLKL), resulting in plasma membrane damage and necroptosis [7]. CASP8 activity is controlled by a catalytically inactive paralogue, the cellular FLICE-like inhibitory protein (cFLIP). Its long isoform (cFLIP_L_) binds CASP8 and was considered to mainly affect CASP8-mediated apoptosis without interfering with the capability of CASP8 to inhibit necroptosis [8, 9]. Recent studies using novel genetic models further indicated that cFLIP is a decisive factor in regulating apoptosis, necroptosis and inflammatory signalling induced by CASP8 [10, 11]. The short cFLIP isoform (cFLIP_S_) perturbs CASP8 filament structure and inhibits its enzymatic activity. The ratio of CASP8 to cFLIP_L_ is increasingly viewed as a determining factor controlling pro-apoptotic function of CASP8 by limiting CASP8 homodimer formation. CHX is believed to promote TNF-induced TRADD-FADD-CASP8 complex (complex IIa) formation and apoptosis mainly by reducing the amount of cFLIP_L_ [6, 12].

TNF is a major inflammatory cytokine that was first identified for its ability to induce rapid haemorrhagic necrosis of tumours including melanomas [13, 14]. Further efforts to harness the anti-tumour activity of TNF, however, led to some paradoxical tumour-promoting activities of TNF raising the question whether TNF serves as a “target” or “therapeutic” in cancer [15]. In our study, we aimed to show that TNF serves as a key tissue surveillance mechanism which induces tumour cell apoptosis and blocks melanoma growth when cFLIP is not expressed.

## Materials and methods

### Cell culture

B16F10 cells (freshly obtained from ATCC) and human melanoma cell lines [16–19] were cultured in Dulbeccos Modified Eagle´s Medium (DMEM; Bio&SELL, Feucht, Germany) containing 10% heat-inactivated fetal bovine serum (FCS; Biowest, Nuaillé, France), 100 U/ml penicillin and 100 µg/ml streptomycin (Bio&SELL) at 37 °C and 5% CO_2_. All cells were regularly tested for mycoplasma contamination.

The human B cell line L1309 [20, 21] and murine dermal fibroblasts (MDFs), isolated from tails of wt and *Ripk3*^*-/-*^ C57BL/6 N mice [22], were used as control for human and murine RIPK3 protein expression, respectively.

### Generation of stable knockout B16F10 cells

Stable *Casp8*, *Birc2/3* (encoding cIAP1/2, in the following *cIap1/2*), *Ripk1* or *Cflar* (encoding cFLIP, in the following *cFlip*) B16F10 knockout cells were generated using a modified Nature protocol [23]. Single guide RNAs (sgRNAs) were designed in silico (Table S1) and cloned into the pSpCas9(BB)-2A-GFP (PX458) vector (provided by Feng Zhang, Addgene #48138). Correct insertion was validated by genomic DNA sequencing.

B16F10 cells (3 × 10^6^) were transfected overnight with PX458 vectors using 300 µl OptiMEM (Gibco, Thermo Fisher Scientific, Dreieich, Germany) containing 3 µg vector DNA and 13,5 µl polyethylenimine (PEI) (Polysciences Europe GmbH, Hirschberg an der Bergstrasse, Germany). After 24 h, GFP positive cells were sorted by FACS (BD Influx™; BD Biosciences, Franklin Lakes, NJ, USA) and cultured in 6-well plates. After sufficient growth, single cells were plated in 96-well plates and expanded. Knockout efficiency was validated by immunoblotting and sequencing. For the *cIap1/2* double-knockout cells, *cIap2* was deleted in a *cIap1* knockout clone and knockout was confirmed by sequencing due to the lack of a cIAP2 antibody [17, 24, 25].

### Subcutaneous injection of B16F10 cells

C57BL/6 N mice were received from the CECAD animal facility and *Tnf*^*KO/KO*^ mice [26] from the CRC1403 central repository (Cologne University, Germany). All mouse experiments were approved by local authorities (LAVE, North Rhine-Westphalia, Germany) and conducted in accordance with the German animal protection law. Mice were housed in the animal facility of the University of Cologne under standard pathogen-free conditions with a 12 h light/dark cycle. Food and water were provided ad libitum. A sample size of 10 mice per group was calculated using G*Power based on tumour size on day 10 and a biologically relevant 50% tumour reduction (effect size 1.214; control group: mean 750 cm^3^ and s.d. 350 cm^3^, knockout group: mean 325 cm^3^ and s.d. 325 cm^3^; alpha error probability 0,05; power: 0.8). Randomisation and blinding were not performed. Experiments were conducted in two independent rounds with five mice per group. Mice were 6–10 weeks old at injection.

All cells were tested mycoplasma-negative prior to injection. Wt or knockout B16F10 cells (0.5×10^6^ cells in 100 µl PBS) were injected subcutaneously into the right flank of C57BL/6 N or *Tnf*^*KO/KO*^ mice. Tumour growth was measured daily in two dimensions. Mice were euthanized by cervical dislocation on day 13 or when reaching the endpoint (tumour diameter >15 mm). Tumour volume was calculated as (length × width^2^)/2) [27–29]. Tumour measurements and mouse sex are summarised in Table S2. Mann–Whitney U test was used to assess differences in tumour volume due to non-normal distribution and/or unequal variance.

### H&E staining of tumours

Cryosections (8 µm) of B16F10 tumours were cut with a microtome and mounted on glass. Slides were incubated for 10 min in tap water, stained with Haematoxylin (Waldeck, Münster, Germany) for 2 min, washed, and further incubated for 15 min in tap water followed by 1 min in demineralised water. Sections were stained for 1 min in Eosin (Leica, Nussloch, Germany), dehydrated through graded ethanol and xylol, and mounted with Tissue-Tek (Sakura, Umkirch, Germany). Slides were scanned using a NanoZoomer S360 digital slide scanner and analysed with NDP.view2 software (Hamamatsu, Japan).

### Immunoblot analysis

After treatment with the indicated substances, cells were washed with DPBS (Gibco) and homogenised in lysis buffer (20 mM Tris-HCl pH 7.5, 135 mM NaCl, 1.5 mM MgCl_2_, 1 mM EGTA, 1% Triton X-100, 10% glycerol, protease/phosphatase inhibitors (Roche, Basel, Switzerland)) on the plate. After 20 min incubation on ice, the lysates were centrifuged for 15 min at 20,000 x *g* at 4 °C. Protein concentration of the soluble fraction was performed with Pierce™ BCA Protein Assay Kit (Thermo Fisher Scientific). Proteins were separated by SDS-PAGE and transferred to a PVDF (BioRad, Neuried, Germany) membrane. Antibodies used for protein detection are listed in Table S3. Uncropped Western blots can be found in *Supplementary Materials*.

### Cell death analysis

On the day before treatment, 8000 cells per well were plated on a 96-well plate. Cells were treated with the following substances in the presence of Draq7 (Biostatus, Shepshed, UK) or Sytox Green (Invitrogen, Schwerte, Germany): 10 ng/ml (0.578 nM) murine TNF (R&D Systems, Minneapolis, MN, USA), 10 ng/ml (0,575 nM) human TNF (PeproTech, Hamburg, Germany), 20 µM emricasan (Biozol, Hamburg, Germany), 20 µM birinapant (Biozol), 20 µM Nec1S (MedChemExpress, Monmouth Junction, NJ, USA), 1 µg/ml (3.55 µM) cycloheximide (Sigma-Aldrich, Steinheim, Germany), 200 ng/ml (6.25 nM) SUPERFASLIGAND^®^ (FasL, Enzo Life Sciences, Lörrach, Germany), 400 ng/ml (15,38 nM) SuperKillerTRAIL^®^ (Enzo Life Sciences). Cell death was measured with an IncuCyte S3 (Sartorius, Göttingen, Germany) every 2 h within 24 h. After 24 h, 200 µg/ml (162.69 µM) digitonin (Calbiochem, Darmstadt, Germany) was added to induce 100% cell death. Percent cell death was calculated relative to 100% cell death.

### cFLIP and Casp8 knockdown

The human melanoma cell lines BLM, SK-MEL-8, MaMel86a, and A375 [16, 18], and the *cFlip*^*KO/KO*^ B16F10 cells were seeded at 70% confluency, and the day after, transfected with siRNAs targeting *cFLIP* (1:1 mix of GGGACCUUCUGGAUAUUUU and AACUGCUCUACAGAGUGAGGC), *cFLIP*_*L*_ (GAGCUUCUUCGAGACACCU), *cFLIP*_*S*_ (CACCCUAUGCCCAUUGUCC) [30], *Casp8* (GUGAAUGGAACCUGGUAU), or the non-targeting control siRNA (siScr, GGAUUACUUGAUAACGCUAUU) using RNAiMAX (Invitrogen) according to the manufacturer’s instructions. After 24 h, transfection medium was removed, and cells were treated with 10 ng/ml TNF (human: PeproTech, murine: R&D Systems) +/− 20 µM emricasan (Biozol) for 4 h (Western blot) or 24 h (IncuCyte cell death analysis).

## Results

### Melanoma cells resist TNF-induced apoptosis

**Fig. 1:**
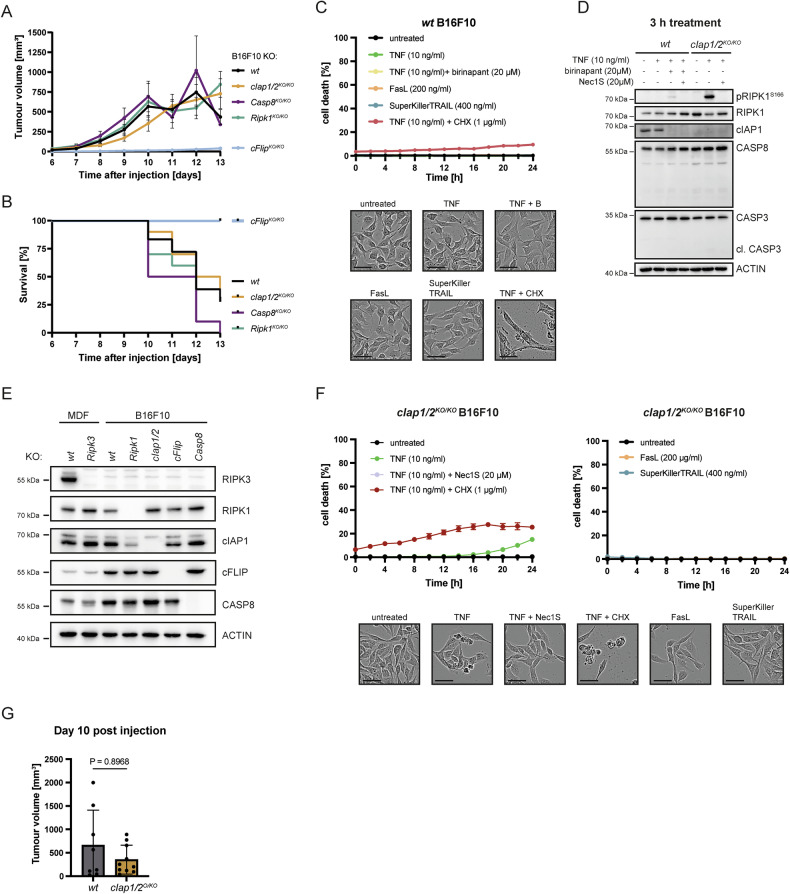
Melanoma cells resist TNF-induced apoptosis. **A** 5×10^5 ^*wt, cIap1/2*^*KO/KO*^*, Casp8*^*KO/KO*^*, Ripk1*^*KO/KO*^*, or cFlip*^*KO/KO*^ B16F10 cells were subcutaneously injected into the right flank of C57BL/6 N mice. Tumour volume was measured in two dimensions every day and calculated as (length × width^2^)/2. Mice were euthanised via cervical dislocation at day 13 or when reaching the endpoint (tumour size of >15 mm in diameter). Tumour volume is shown as mean +/- s.e.m., *n* = 18 mice for wt B16F10 and *n* = 10 for knockout B16F10. Reduction in mean tumour volume on days 11 and 13 is attributable to the sacrifice of mice on the preceding days upon reaching the ethical endpoint. **B** Kaplan-Meier curve showing survival of mice from (**A**). **C** *wt* B16F10 cells were treated in vitro as indicated and cell death was measured for 24 h with an IncuCyte S3 live cell imager. Where indicated, birinapant was pretreated for 1 h to induce cIAP1/2 degradation. Top: Data are mean ± s.d., *n* = 3 technical replicates. Bottom: representative pictures of cells treated as indicated; scale bar = 50 µm. B: birinapant. Data is representative of three independent experiments. **D** w*t* and *cIap1/2*^*KO/KO*^ B16F10 cells were treated for 3 h as indicated and phosphorylation of RIPK1 as well as Caspase-3 and -8 cleavage were assessed via Western blot. Where indicated, birinapant was pretreated for 1 h to induce cIAP1/2 degradation. **E** Western blot analysis of lysates from B16F10 cells validating knockout of *Ripk1*, *cIap1/2*, *cFlip*, and *Casp8*, as well as absence of RIPK3 expression compared to murine dermal fibroblasts (MDFs). **F** *cIap1/2*^*KO/KO*^ B16F10 cells were treated in vitro as indicated and cell death was measured over 24 h with an IncuCyte S3 live cell imager. Top: Data are mean ± s.d., *n* = 3 technical replicates. Bottom: Representative pictures of cells, treated as indicated, scale bar = 50 µm. Data are representative of three independent experiments. **G** 5×10^5 ^*wt* or *cIap1/2*^*KO/KO*^ B16F10 cells were injected *s.c*. into the right flank of C57BL/6 N mice. Bar chart showing the tumour volume on day 10 after injection calculated as (length × width^2^)/2. Data is presented as mean +/- s.d., dots represent individual mice (*n* = 8–10 per group). Significance was tested using Mann-Whitney test.

To examine the role of extrinsic apoptosis in melanoma progression, we employed a syngeneic melanoma mouse model, utilising immune-competent mice bearing B16F10 cutaneous melanomas [17, 27–29]. Upon subcutaneous injection, B16F10 cells formed palpable tumours and grew subcutaneously already within 7 days (Fig. 1A). Mice were either euthanised based on the humane endpoint (tumour diameter > 15 mm) or sacrificed on day 13 (Fig. 1B). Notably, as some mice with high tumour volumes had to be euthanised ahead of the scheduled endpoint on day 13, the respective animals/tumours were not included in our analysis on the following day and the mean tumour volume appeared accordingly reduced.

Further analysis, examining the susceptibility of B16F10 cells to DRs activation, revealed that these cells resist treatments involving FAS, TRAIL-R and TNF-R1. Additional exposure to CHX, involving the cytosolic complex consisting of TRADD-FADD-CASP8 (complex IIa), induced only 5–10% cytotoxicity (Fig. 1C). Treatment of cells with IAP antagonists potentiates TNF-induced apoptosis by rapidly inducing proteasomal degradation of cIAPs [31, 32] followed by dimerisation of RIPK1, autophosphorylation at serine 166 (S166), and formation of the cytosolic complex II, involving FADD and CASP8, ultimately leading to CASP8 activation and apoptosis [33, 34]. The IAP antagonist birinapant, however, failed to promote cytotoxicity in response to TNF, collectively indicating that B16F10 cells robustly resist extrinsic apoptosis.

Western blot (WB) analysis of B16F10 cellular lysates showed that birinapant treatment induced cIAP1 degradation and autophosphorylation of RIPK1, yet neither CASP8 nor CASP3 activation/processing could be detected 3 hours (h) post-treatment (Fig. 1D). At later time points (18 h post treatment), RIPK1 cleavage and mild CASP3 processing could be detected in cellular lysates of B16F10 cells treated with TNF/birinapant (Fig. S1A). CASP8 processing was, however, not evident. This is in line with a recent study demonstrating that the non-cleavable CASP8(D387A) within the cytosolic complex consisting of RIPK1-FADD-CASP8 (complex IIb) efficiently cleaves RIPK1 and CASP3 [10]. Intriguingly, CASP8-mediated cleavage of RIPK1 and CASP3 was less efficient in cells lacking cFLIP, indicating that the non-cleavable CASP8(D387A) requires cFLIP in order to process down-stream targets such as RIPK1 and CASP3. Furthermore, RIPK1 kinase activity and its autophosphorylation at S166 is known to serve as an amplification loop for clustering further RIPK1 molecules and further accumulation of the complex IIb, which, in turn, potentiates more CASP8 activity [33, 35]. Accordingly, the RIPK1 kinase inhibitor Nec1S reduced the cleavage of RIPK1 and CASP3 (Fig. S1A).

Collectively, these observations indicated that targeting cIAP1/2 may not provide a valuable therapeutic option for treating melanoma. Although no direct cytotoxic activity toward tumour cells could be measured, our previous study showed that IAP antagonists can inhibit melanoma growth in mice [29]. The anti-tumour activity of IAP antagonist was, however, based on melanoma vascular destruction very similar to the initial observations using TNF as a therapeutic in cancer treatment, leading to tumour haemorrhagic necrosis due to TNF-induced vascular toxicity [15]. To specifically elucidate the role of cIAPs and RIPK1 kinase activity-mediated cytotoxicity in melanoma in vivo, we generated B16F10 cell lines lacking both cIAP1 and cIAP2 (*cIap1/2*^*KO/KO*^ B16F10 cell line) by using CRISPR/Cas9 gene editing (Figs. 1E and S1B).

Similar to wildtype cells treated with birinapant, lack of cIAP1 and cIAP2 in B16F10 cells could not efficiently promote cytotoxicity in response to TRAIL, FAS-L, or TNF (Fig. 1F). A minority of *cIap1/2*^*KO/KO*^ B16F10 cells ( ~ 15%) died in response to TNF after 24 h. WB analysis showed that *cIap1/2*^*KO/KO*^ B16F10 cell strongly accumulated autophosphorylated RIPK1, but failed to potently induce caspase activation/processing (Figs. 1D and S1A). In line, lack of cIAP1/2 in B16F10 cells did not alter melanoma growth in mice (Fig. 1A–B and G). These observations collectively indicate that activation of RIPK1 cannot potentiate tumour cell apoptosis, thus does not interfere with tumour growth in vivo.

### Melanoma cells resist TNF-induced necroptosis and grow in mice independently of RIPK1-mediated cell death signalling

**Fig. 2:**
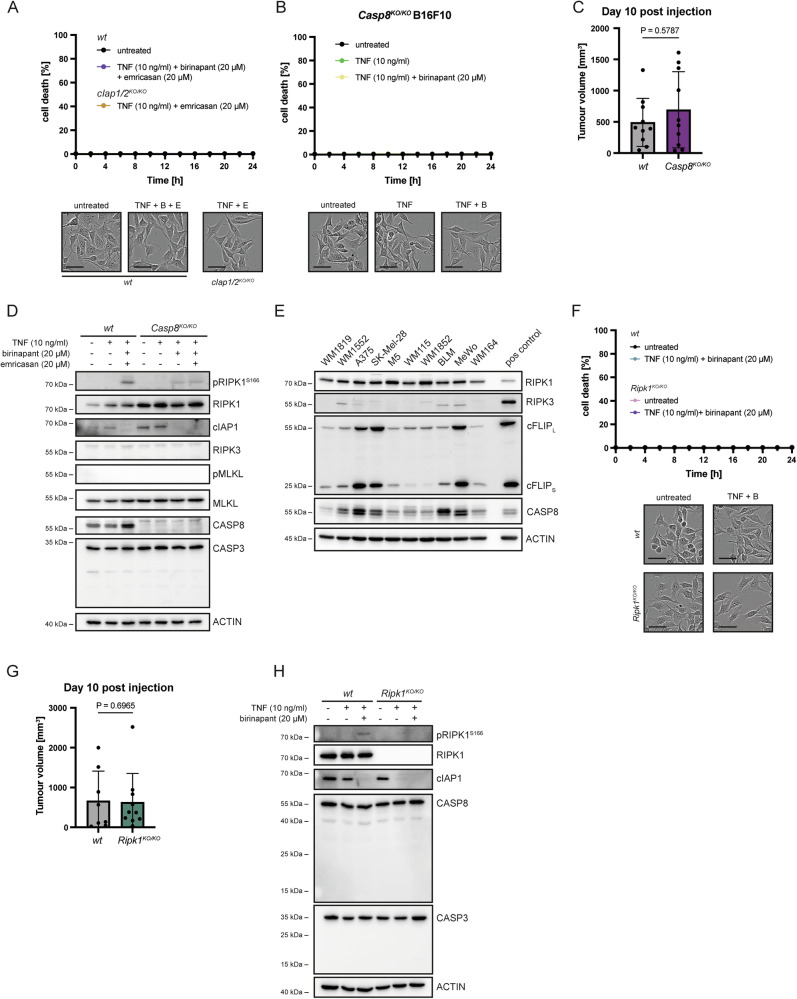
Melanoma cells resist TNF-induced necroptosis and grow independently of RIPK1-mediated cell death signalling in mice. **A** w*t* and *cIap1/2*^*KO/KO*^ B16F10 cells were treated in vitro as indicated and cell death was measured for 24 h with an IncuCyte S3 live cell imager. Where indicated, birinapant was pretreated for 1 h to induce cIAP1/2 degradation. Top: Data are mean ± s.d., *n* = 3 technical replicates. Bottom: representative pictures of cells, treated as indicated, scale bar = 50 µm. B: birinapant, E: emricasan. Data is representative of three independent experiments. **B** *Casp8*^*KO/KO*^ B16F10 cells were treated in vitro as indicated and cell death was measured for 24 h with an IncuCyte S3 live cell imager. Where indicated, birinapant was pretreated for 1 h to induce cIAP1/2 degradation. Top: Data are mean ± s.d., *n* = 3 technical replicates. Bottom: representative pictures of cells, treated as indicated, scale bar = 50 µm. B: birinapant. Data are representative of three independent experiments. **C** 5 × 10^5 ^*wt* or *Casp8*^*KO/KO*^ B16F10 cells were injected *s.c*. into the right flank of C57BL/6 N mice. Bar chart showing the tumour volume on day 10 after injection, calculated as (length × width^2^)/2. Data is presented as mean +/- s.d., dots represent individual mice (*n* = 10 per group). Significance was tested using Mann-Whitney test. **D** Western blot analysis of *wt* and *Casp8*^*KO/KO*^ B16F10 cells treated for 3 h with TNF, emricasan and birinapant (pretreatment for 1 h). Western blot is representative of three independent experiments. **E** Western blot analysis of different human melanoma cell lines showing RIPK1, RIPK3, cFLIP and CASP8 protein expression. Positive control for RIPK3 expression: human L1309 B cell line. **F** *wt* and *Ripk1*^*KO/KO*^ B16F10 cells were treated in vitro as indicated and cell death was measured for 24 h with an IncuCyte S3 live cell imager. Where indicated, birinapant was pretreated for 1 h to induce cIAP1/2 degradation. Top: Data are mean ± s.d., *n* = 3 technical replicates. Bottom: representative pictures of cells treated as indicated, scale bar = 50 µm. Data is representative of three independent experiments. **G** 5 × 10^5 ^*wt* or *Ripk1*^*KO/KO*^ B16F10 cells were injected *s.c*. into the right flank of C57BL/6 N mice. Bar chart showing the tumour volume on day 10 after injection, calculated as (length × width^2^)/2. Data is presented as mean +/- s.d.; dots represent individual mice (*n* = 8–10 per group). Significance was tested using Mann-Whitney test. **H** Western blot analysis of *wt* or *Ripk1*^*KO/KO*^ B16F10 cells treated with TNF and birinapant (pretreatment for 1 h). Western blot is representative of three independent experiments.

When CASP8 activity is blocked, activation/autophosphorylation of RIPK1 normally leads to a direct interaction of RIPK1 with RIPK3, autophosphorylation and activation of RIPK3, and phosphorylation of MLKL causing necroptosis [36, 37]. B16F10 cells treated with birinapant or lacking cIAP1/2 accumulated autophosphorylated RIPK1 and failed to activate CASP8. Yet, they did not die in response to TNF (Fig. 1). We were also not able to potentiate TNF-induced necroptosis when we exposed cells to the pan-caspase inhibitor emricasan (Fig. 2A). To examine whether the potentiation of necroptosis by removing CASP8, the key inhibitor of necroptosis, can interfere with melanoma growth in vivo, we established *Casp8*^*KO/KO*^ B16F10 cell lines (Figs. 1E and S2A). Similar to B16F10 cells treated with emricasan, we were not able to induce necroptosis in *Casp8*^*KO/KO*^ B16F10 cells lacking CASP8 expression (Fig. 2B). In line, the lack of CASP8 did not interfere with in vivo melanoma growth in mice (Figs. 1A-B and 2C), collectively suggesting that necroptosis is robustly blocked in B16F10 cells.

WB analysis of cellular lysates showed that TNF/birinapant/emricasan treatment induced RIPK1 autophosphorylation. However, no phosphorylated MLKL was detectable (Fig. 2D). Further, while B16F10 cells express MLKL, RIPK3 was barely detectable, suggesting that their inability to undergo necroptosis is due to the lack of RIPK3 expression (Figs. 2D and 1E). This is consistent with previous studies showing that lack of RIPK3 expression predicts necroptosis resistance in malignant melanoma [38]. Similarly, RIPK3 was not detectable in cellular lysates derived from human melanoma cell lines (Fig. 2E).

Our results collectively indicated that cell death machineries involving RIPK1 kinase activity are inefficient in killing B16F10 melanoma cells. In addition to its kinase activity, RIPK1 scaffold represents a central element in both CASP8-mediated apoptosis and RIPK3-mediated necroptosis. Accordingly, RIPK1 knockout leads to embryonic lethality in mice by unleashing apoptotic and necroptotic signalling [39–41]. Based on this, RIPK1 degraders are currently developed and considered as a potent anti-cancer treatment strategy, particularly in cancer immunotherapy [42]. To examine the role of RIPK1 scaffold, we generated a *Ripk1*^*KO/KO*^ B16F10 cell line (Figs. 1E and S2B). *Ripk1*^*KO/KO*^ B16F10 cells resisted TNF/birinapant treatment (Fig. 2F) and potently grew in mice (Figs. 1A–B and 2G). WB analysis did not show any activation of caspases (Fig. 2H), indicating that B16F10 cells resist apoptosis. Whereas the incapability to undergo necroptosis was caused by lack of RIPK3, the responsible molecular mechanism leading to the failure of caspase activation remained unclear.

### cFLIP confers resistance to TNF and promotes melanoma growth in mice

**Fig. 3:**
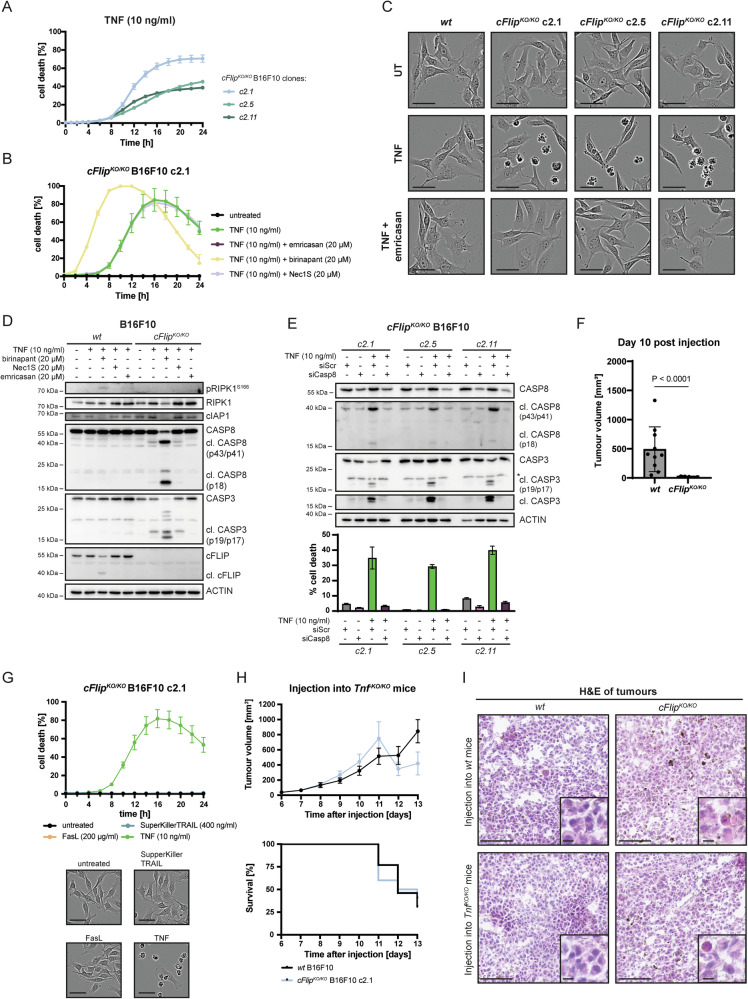
cFLIP confers resistance to TNF and promotes melanoma growth in mice. **A** *cFlip*^*KO/KO*^ B16F10 cells were treated in vitro with 10 ng/ml TNF and cell death was measured for 24 h with an IncuCyte S3 live cell imager. Where indicated, birinapant was pretreated for 1 h to induce cIAP1/2 degradation. Data are mean ± s.d., *n* = 3 technical replicates. Data is representative of three independent experiments. **B** *cFlip*^*KO/KO*^ B16F10 cells were treated in vitro as indicated and cell death was measured for 24 h with an IncuCyte S3 live cell imager. Where indicated, birinapant was pretreated for 1 h to induce cIAP1/2 degradation. Data are mean ± s.d., *n* = 3 technical replicates. Data is representative of three independent experiments. **C** Representative pictures of cells treated for 24 h as in (**A** and **B**). **D** Western blot analysis of *wt* and *cFlip*^*KO/KO*^ B16F10 cells treated for 3 h with TNF and emricasan, Nec1S or birinapant (pretreatment for 1 h). Western blot is representative of three independent experiments. **E** Three independent *cFlip*^*KO/KO*^ B16F10 cell clones were transfected for 24 h with a scrambled siRNA (siScr) or siRNA targeting CASP8 (siCasp8) followed by treatment with TNF. Top: Western blot analysis after 3 h treatment with TNF. Bottom: cell death analysis using an IncuCyte S3 live cell imager after 24 h treatment. Data are mean ± s.d., *n* = 3 technical replicates per independent clone. **F** 5 × 10^5 ^*wt* or *cFlip*^*KO/KO*^ B16F10 cells were injected *s.c*. into the right flank of C57BL/6 N mice. Bar chart showing the tumour volume on day 10 after injection, calculated as (length × width^2^)/2. Data is presented as mean +/- s.d., dots represent individual mice (*n* = 10 per group). Significance was tested using Mann-Whitney test. **G** *cFlip*^*KO/KO*^ B16F10 cells were treated in vitro as indicated and cell death was measured for 24 h with an IncuCyte S3 live cell imager. Data are mean ± s.d., *n* = 3 technical replicates. Data are representative of three independent experiments. **H** 5 × 10^5 ^*wt* or *cFlip*^*KO/KO*^ B16F10 cells were injected *s.c*. into the right flank of *Tnf*^*KO/KO*^ mice. Tumour volume was measured in 2 dimensions every day and calculated as (length × width^2^)/2. Mice were euthanised via cervical dislocation at day 13 or when reaching the endpoint (tumour size of >15 mm in diameter). Top: Tumour volume is shown as mean +/- s.d., *n* = 10 mice. Bottom: Kaplan-Meier curve showing percent survival of mice. **I** Representative H&E stainings of *wt* and *cFlip*^*KO/KO*^ tumours, dissected from C57BL/6 N and *Tnf*^*KO/KO*^ mice. Scale bar: 100 µm and 10 µm for magnification.

cFLIP is a catalytically inactive paralogue of CASP8 which, upon direct binding, controls CASP8 activity. cFLIP has been shown to be highly expressed in human melanoma [43] and its transient downregulation was sufficient to sensitise human melanoma cells to TRAIL- and FasL-mediated apoptosis [44]. To investigate the role of cFLIP in B16F10 melanoma, we established *cFlip*^*KO/KO*^ B16F10 cell line lacking cFLIP expression (Figs. 1E and S3A). *cFlip*^*KO/KO*^ B16F10 cell lines were highly susceptible to TNF (Fig. 3A). Emricasan efficiently inhibited TNF-induced cell death in *cFlip*^*KO/KO*^ B16F10 cells, indicating that TNF induces caspase-dependent cell death when cFLIP is not expressed (Fig. 3B–C). In contrast, RIPK1 kinase inhibitor Nec1S did not alter TNF-induced cytotoxicity. However, exposure to birinapant, which promotes RIPK1-mediated apoptosis, very efficiently facilitated TNF-induced cell death in *cFlip*^*KO/KO*^ B16F10 cells, indicating that cFLIP also inhibits TNF-induced apoptosis involving RIPK1 (Figs. 3B, S3B). Consistent with our cell death analysis, WB showed that TNF treatment rapidly induced caspase processing in *cFlip*^*KO/KO*^ B16F10 cells at an early time point (3 h post-treatment), which was further strongly enhanced by birinapant co-treatment. Nec1S could diminish the effect of birinapant, but could not completely abrogate caspase activation in *cFlip*^*KO/KO*^ B16F10 cells exposed to TNF (Fig. 3D). The central role of CASP8 in the susceptibility of *cFlip*^*KO/KO*^ B16F10 cells to TNF was further confirmed by implementing a CASP8-specific knockdown. CASP8 downregulation completely diminished TNF-induced cell death and caspase activation in *cFlip*^*KO/KO*^ B16F10 cells (Figs. 3E, S3C).

In line with their increased apoptotic propensity, *cFlip*^*KO/KO*^ B16F10 cells failed to grow in mice and barely developed subcutaneous tumours up to 13 days (Figs. 1A–B and 3F), underscoring the central role of cFLIP and the inhibition of extrinsic apoptosis in B16F10 melanoma tumour growth. Notably, unlike TNF, neither FasL nor TRAIL could induce cell death in *cFlip*^*KO/KO*^ B16F10 cells likely due to the lack or low expression of the respective DRs (Fig. 3G). To examine the role of TNF in controlling melanoma growth in mice, we examined the subcutaneous growth of B16F10 melanoma cells in *Tnf*^*KO/KO*^ mice [26]. *cFlip*^*KO/KO*^ B16F10 cells competently grew subcutaneously in *Tnf*^*KO/KO*^ mice and formed palpable tumours already 8 days post-injection (Fig. 3H). Histological analysis of B16F10 and *cFlip*^*KO/KO*^ B16F10 tumours in wildtype and *Tnf*^*KO/KO*^ mice did not reveal gross alteration of tumour cell architecture (Fig. 3I). These data collectively demonstrated that cFLIP supports the growth of B16F10 melanoma in mice by specifically inhibiting TNF-induced apoptosis.

### cFLIP controls susceptibility of human melanoma cells to TNF

**Fig. 4:**
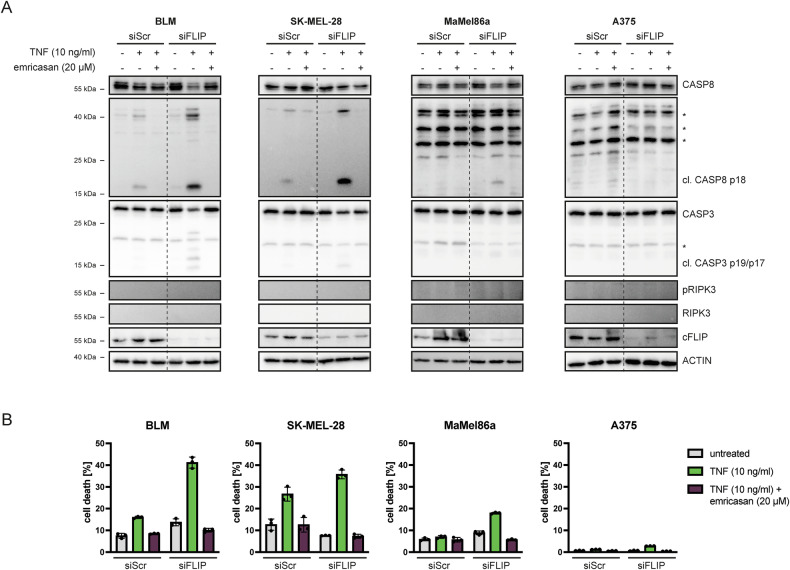
cFLIP controls susceptibility of human melanoma cells to TNF. **A** cFLIP knockdown was induced in BLM, SK-MEL-28, MaMel86a and A375 human melanoma cell lines via siRNAs, and cells were subsequently treated with TNF +/- emricasan for 4 h. Cleavage of CASP3 and CASP7 was assessed via Western blot. Data are representative of two independent experiments. **B** Cell death assessment via IncuCyte live cell imager of cells treated as in (**A**) for 24 h. Data is shown as mean +/- s.d., *n* = 3 technical replicates. Data are representative of two independent experiments.

Previous studies indicated that cFLIP targeting promotes TRAIL- and FasL-mediated apoptosis in human melanoma cell lines [44]. Our data using mouse melanoma cells showed that cFLIP also inhibits TNF-induced cytotoxicity and supports tumour development in vivo. To assess the role of cFLIP in TNF-induced cytotoxicity in human melanoma, we transiently downregulated cFLIP in human melanoma cell lines [16–18]. WB analysis showed that cFLIP downregulation in BLM, SK-MEL-28 and also partially in MaMel86a promoted TNF-induced caspase activation leading to cell death (Fig. 4A–B). Emricasan treatment potently blocked caspase processing and cell death in response to TNF. These observations were consistent with our data obtained in mouse melanoma cells and further confirmed the lack of necroptosis in melanoma cells when cells were exposed to TNF and emricasan. In line with our observations in B16F10 cells, we could barely detect RIPK3 in human melanoma cell lines (Figs. 2E and 4A). These data demonstrate the anti-tumour activity of TNF in human melanoma cells and underscore the decisive role of cFLIP controlling melanoma responses to TNF. Importantly, while cFLIP downregulation promoted TNF-induced cytotoxicity in BLM, SK-MEL-28 and MaMel86a melanoma cell lines, it failed to impact on TNF-induced caspase activity and cell death in the BRAF-mutant A375 human melanoma cell line (Fig. 4A–B) which has been shown to undergo extrinsic apoptosis in response to IAP antagonist [18, 45]. Independently, long and short c-FLIP isoforms represent the main *CFLAR* gene products that bind and regulate CASP8 activity. Whereas heterodimers of CASP8 and cFLIP_L_ also promote CASP8 activity, which is independent of its autoprocessing [8], cFLIP_S_ perturbs CASP8 filament structure and inhibits its enzymatic activity [46]. In order to examine the role of cFLIP isoforms, we performed specific knockdown of cFLIP_L_ and cFLIP_S_ [30] in human melanoma cell lines and analysed cell death and caspase activation in response to TNF (Fig. S4A-B). Although different melanoma cell lines responded differently to cFLIP knockdown (non-responsive A375, highly responsive SK-MEL-28), specific knockdown of cFLIP_L_ generally promoted TNF-induced cell death and caspase activation similar to cFLIP downregulation (long and short). Specific knockdown of cFLIP_S_ was less efficient in melanoma cell lines and promoted only partial enhancement of TNF-induced cell death.

## Discussion

TNF is a pleiotropic inflammatory cytokine initially described based on its anti-tumour activities [13, 14]. Further evidence, however, indicated that TNF-induced inflammation and even its direct effects on tumour cells may actually be cancer promoting [15]. Particularly, in melanoma, recent studies indicated that TNF reduces the efficacy of melanoma immune surveillance and immunotherapies involving tumour-specific cytolytic T lymphocytes [47]. Furthermore, immune checkpoint inhibitors (ICIs) represent an effective treatment option in patients with advanced melanoma [48, 49]. Elevated plasma TNF in these patients is associated with poorer clinical outcomes for such therapeutic interventions, reinforcing the notion that TNF may dampen immunotherapy efficacy in melanoma [50, 51]. Recent studies substantially increased our understanding of TNF-induced signalling pathways and cellular responses across diverse tissues [52]. Accumulating evidence now shows that, depending on the context, TNF can mediate either pro-survival or pro-death signals, indicating that TNF can be either detrimental or favourable to cancer progression, and that the ultimate outcome might depend on several factors including the amount of TNF, tissue, or the tumour type [53]. In particular, multiple recent studies using pooled loss-of-function screening in cancer by utilizing CRISPR/Cas9 technology have identified various components of the TNF signalling cascade that determine tumour cell killing induced by CD8^+^ CTLs, natural killer (NK) cells, ICI treatment, and CAR-T cells [54]. These findings suggest that targeting components of the TNF signalling pathway may be an innovative approach to increase immunotherapy efficacy. Consistently, we showed that targeting of cFLIP enables TNF to efficiently kill melanoma cells and block melanoma growth in mice. These data provide an enticing prospect to augment immunotherapy responses through promoting TNF cytotoxicity in tumour cells. Importantly, whereas the majority of the recent efforts focused on targeting IAPs and promoting RIPK1-dependent TNF cytotoxicity in cancer (complex IIb), our results here underscore cFLIP as a valuable target to promote anti-tumour activity of TNF by involving both complex IIa and complex IIb.

The design of small molecules that directly bind to cFLIP and thereby promote apoptosis has long been considered as a potent therapeutic strategy for cancer [46]. However, most efforts have failed, as cFLIP shares significant structural similarity to CASP8, making selective inhibition difficult without also blocking CASP8 [55]. An increasing number of studies are employing peptide/compound libraries and currently aim to identify cFLIP-specific binders without affecting CASP8, paving the way for the design of new and effective cFLIP inhibitory molecules that may serve as anticancer agents. One striking observation in our studies was the fact that murine melanoma cells harbouring a cFLIP knockout were not able to grow in vivo because of efficient anti-tumour activity of endogenous TNF. Therefore, our data suggest that therapeutic strategies targeting cFLIP will likely be able to eliminate cancer cells or block cancer growth by activating the body’s immune surveillance mechanism, thus eliminating the need for further treatment. We posit that such an approach can also be an appealing opportunity to enhance immunotherapy responses, which requires further experimental evaluation.

## Supplementary information

Supplementary Information

Supplemental Material - uncropped WBs

Table S1

Table S2

Table S3

## Data Availability

Primary data are available from the corresponding author upon request.
