# Supplementary material for: The anti-tumour activity of TNF in melanoma is determined by cFLIP
Source: Cell Death Dis. 2026 Aug 1;17(1):671. doi: 10.1038/s41419-026-09154-6 (PMC13428747; doi:10.1038/s41419-026-09154-6)
Supplement: Supplementary file 1 — Supplementary Information [file 41419_2026_9154_MOESM1_ESM.docx]

**Supplementary Information**

**Table of contents**

Figure S1…………………………………………………………………………. 2

Figure S2…………………………………………………………………………. 3

Figure S3…………………………………………………………………………. 4

Figure S4…………………………………………………………………………. 5

**
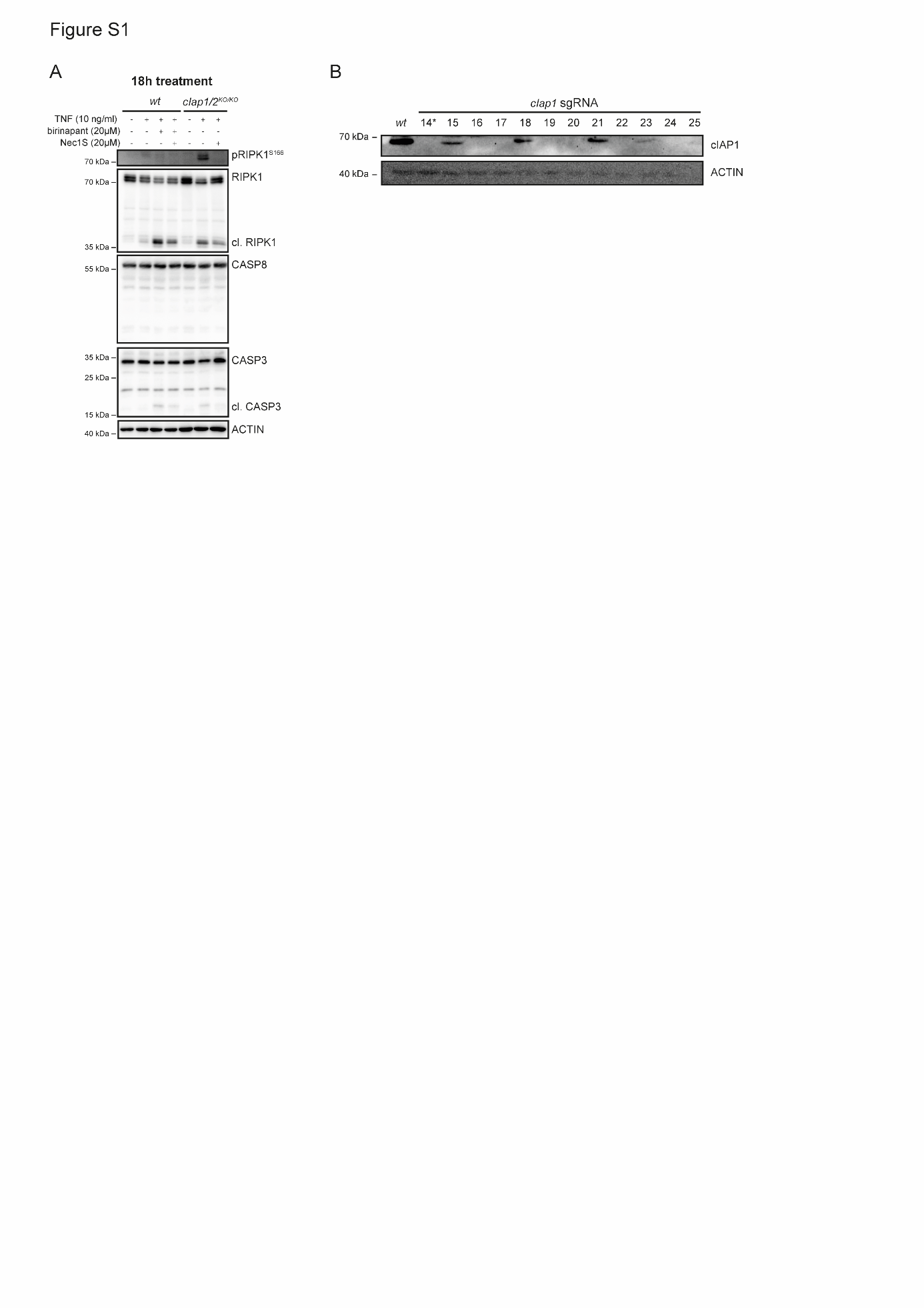
**

**Figure S1. B16F10 cells are resistant to TNF-induced extrinsic apoptosis.**

**A.** Western blot analysis of *wt* and *cIap1/2^KO/KO^* B16F10 cells treated for 18 h as indicated. Where indicated, Birinapant was pretreated for 1 h to induce cIAP1/2 degradation. **B.** Validation of *cIap1* knockout in B16F10 cells by CRIPR/Cas9 in single cell clones via western blot. Clone 14, indicated by *, was used for a subsequent knockout of *cIap2*.


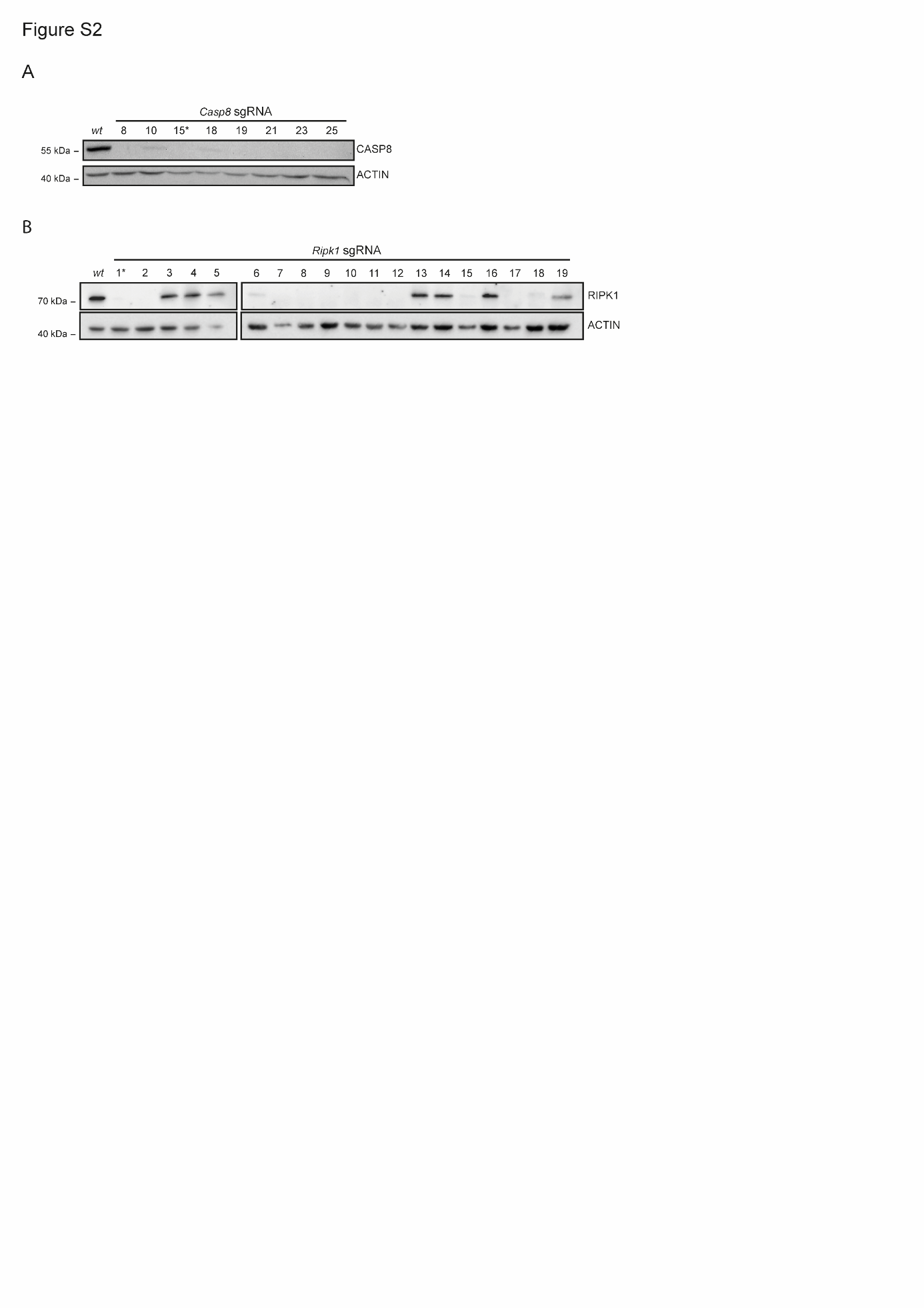


**Figure S2. Validation of *Casp8* and *Ripk1* knockout in B16F10 cells by CRISP/Cas9.**

**A.** Validation of *Casp8* knockout in B16F10 cells by CRIPR/Cas9 in single cell clones via Western blot. Clone 15, indicated by *, was used for experiments. **B.** Validation of *Ripk1* knockout in B16F10 cells by CRIPR/Cas9 in single cell clones via Western blot. Clone 1, indicated by *, was used for experiments.


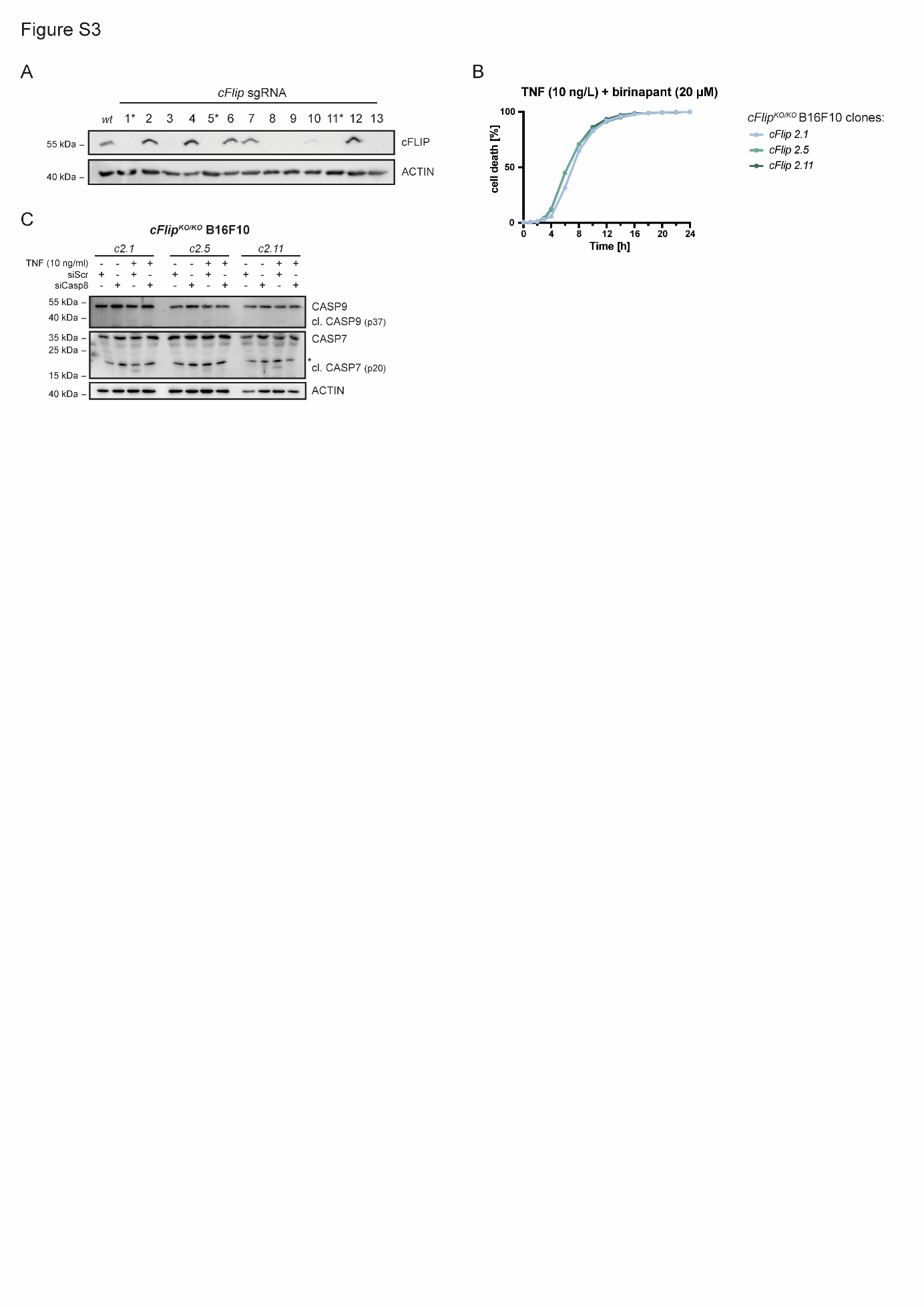


**Figure S3. Knockout of *cFlip* sensitises cells to TNF-induced and RIPK1-dependent apoptosis.**

**A.** Validation of *cFlip* knockout in B16F10 cells by CRIPR/Cas9 in single cell clones via Western blot. Clones 1, 5 and 11, indicated by *, were used for experiments.**B.** *cFlip^KO/KO^* B16F10 cells were treated *in vitro* with TNF and birinapant (1 h pretreatment) and cell death was measured for 24 h with an IncuCyte S3 live cell imager. Data are mean ± s.d., n=3 technical replicates. Data is representative of three independent experiments.**C.** Western blot analysis of three independent *cFlip^KO/KO^* B16F10 cell clones that were transfected for 24h with a scrambled siRNA (siScr) or siRNA targeting CASP8 (siCasp8) followed by 4 h treatment with TNF (see also **Fig. 3E**).


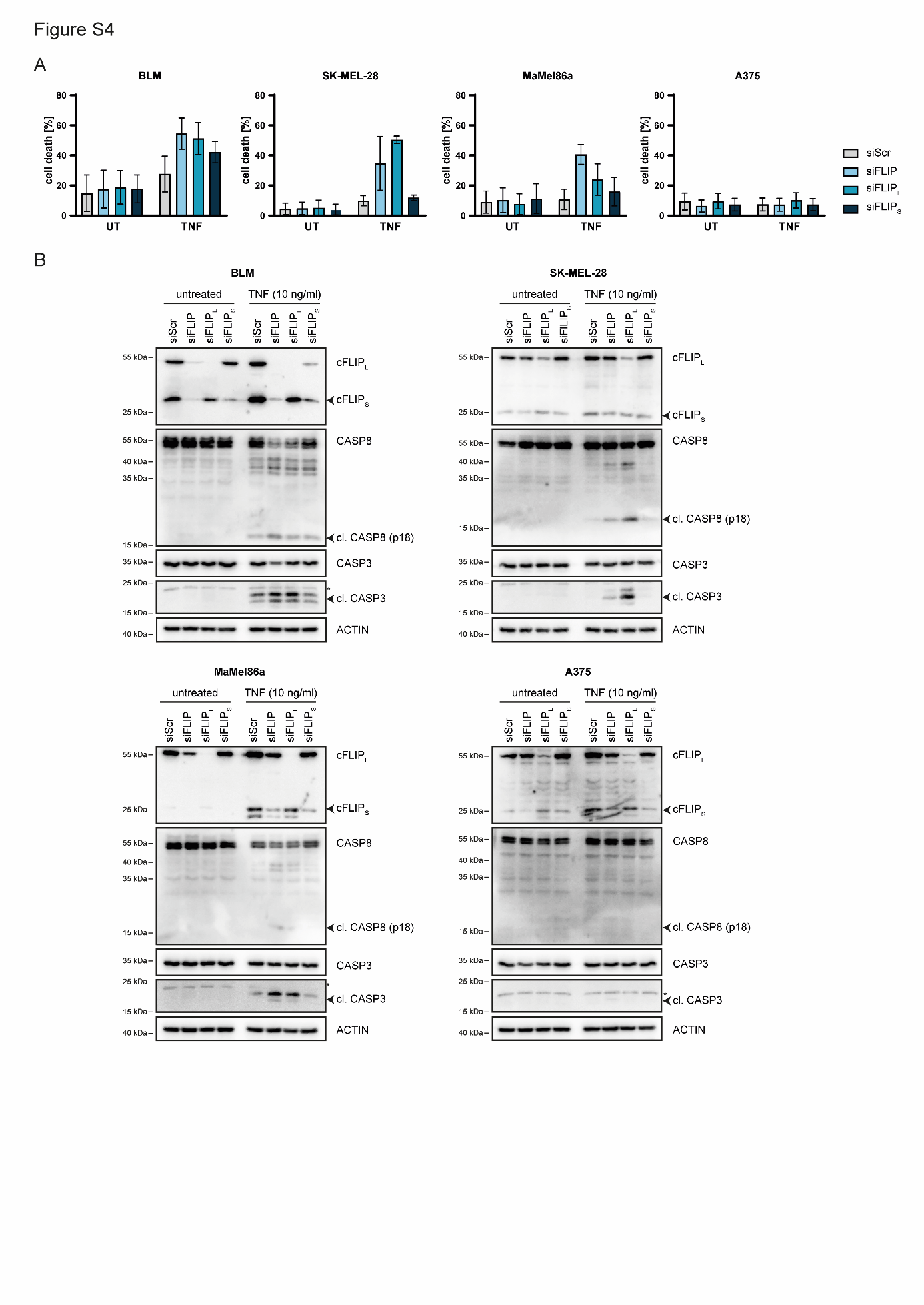


**Figure S4. Specific knockdown of cFLIP short and long isoforms in human melanoma cell lines.**

**A.** Total and isotype-specific cFLIP knockdown was induced in BLM, SK-MEL-28, MaMel86a and A375 human melanoma cell lines via siRNAs and cells were subsequently treated with TNF for 24 h. Cell death was assessment via IncuCyte live cell imager. Data is shown as mean +/- s.d., n=3 biological replicates. **B.** Western blot analysis of cells treated as in (A) for 4 h, assessing knockdown of cFLIP isoforms and cleavage of caspase-3 and -8.
