# Supplementary material for: The anti-tumour activity of TNF in melanoma is determined by cFLIP
Source: Cell Death Dis. 2026 Aug 1;17(1):671. doi: 10.1038/s41419-026-09154-6 (PMC13428747; doi:10.1038/s41419-026-09154-6)
Supplement: Supplementary file 2 — Supplemental Material - uncropped WBs [file 41419_2026_9154_MOESM2_ESM.pdf]

Figure 1D

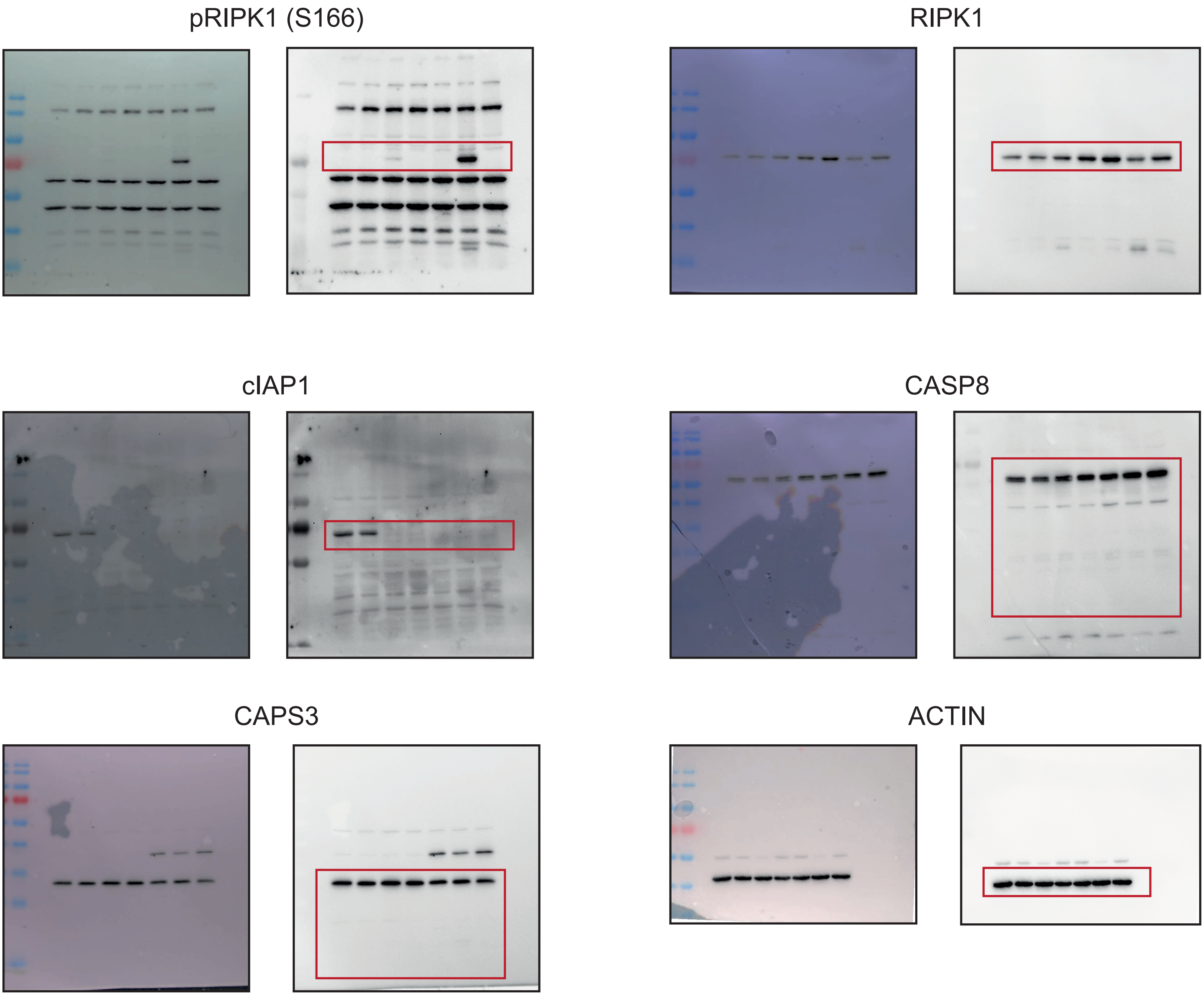

Ladder: PageRuler prestained protein ladder, 10 to 180 kDa, Thermo Fisher Scientific, Cat: 26616
Samples were loaded on different gels, beta actin was detected on one of them as sample processign control.

Figure 1E

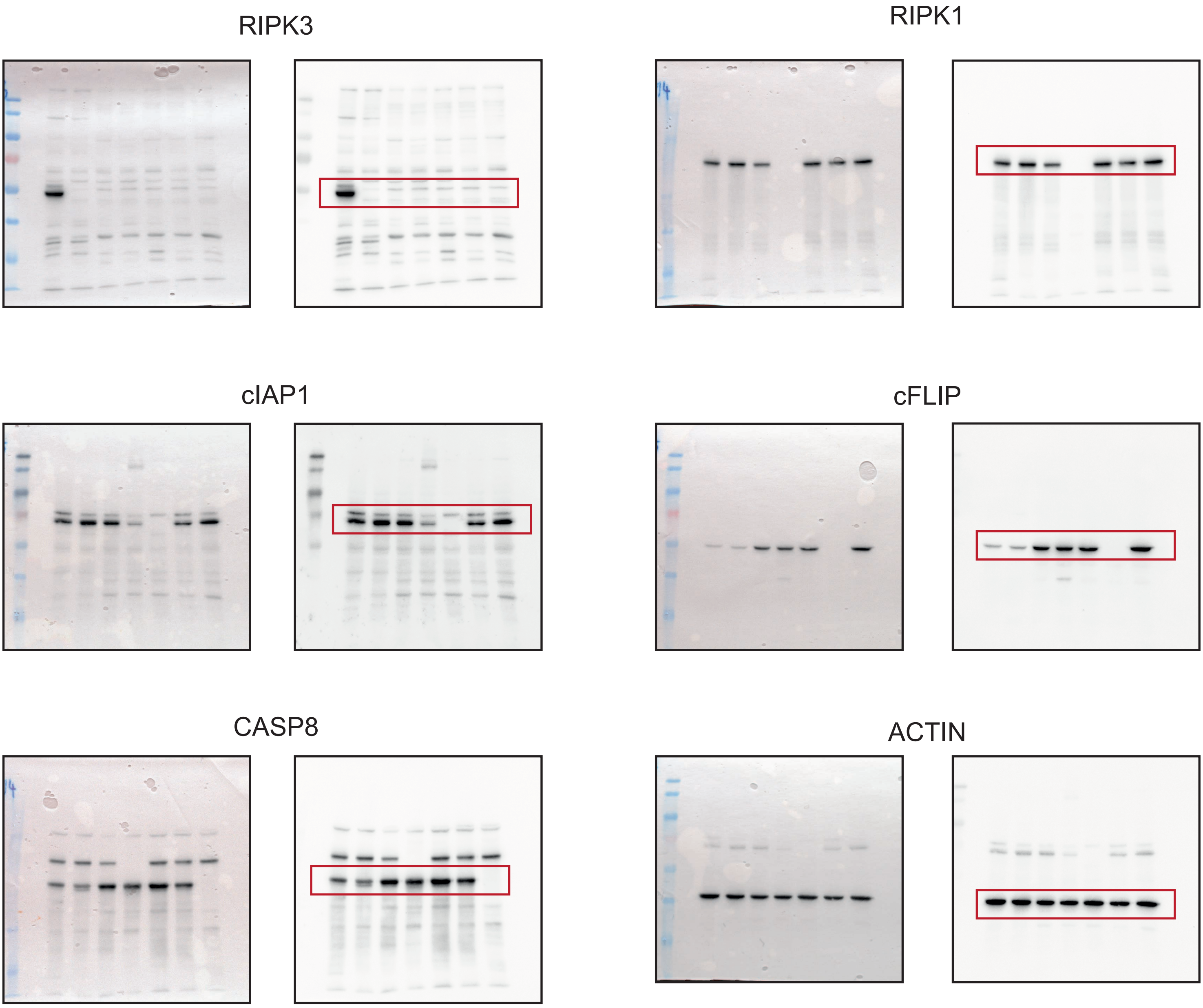

Ladder: PageRuler prestained protein ladder, 10 to 180 kDa, Thermo Fisher Scientific, Cat: 26616
Samples were loaded on different gels, beta actin was detected on one of them as sample processign control.

Figure 2D

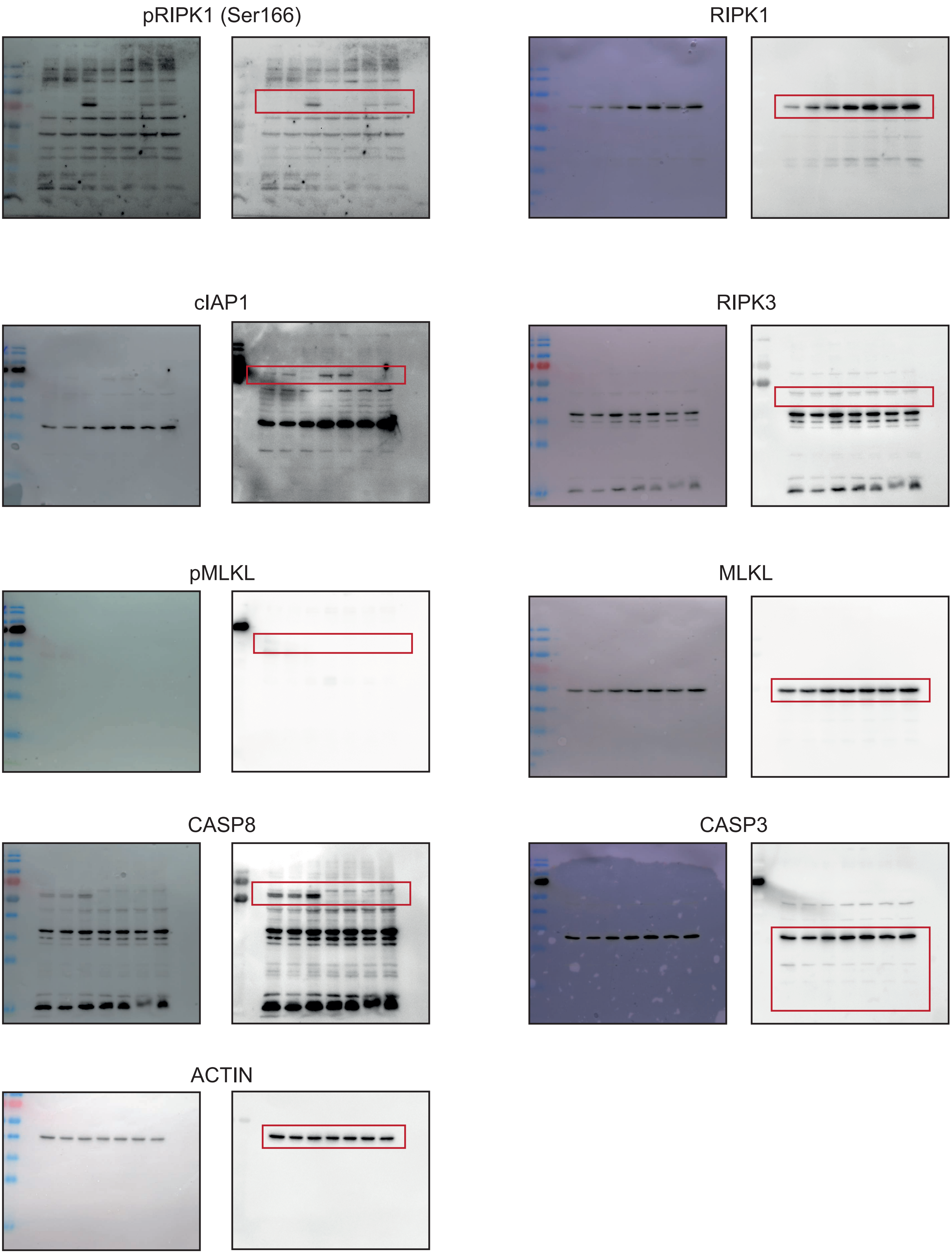

Ladder: PageRuler prestained protein ladder, 10 to 180 kDa, Thermo Fisher Scientific, Cat: 26616
Samples were loaded on different gels, beta actin was detected on one of them as sample processign control.

Figure 2E

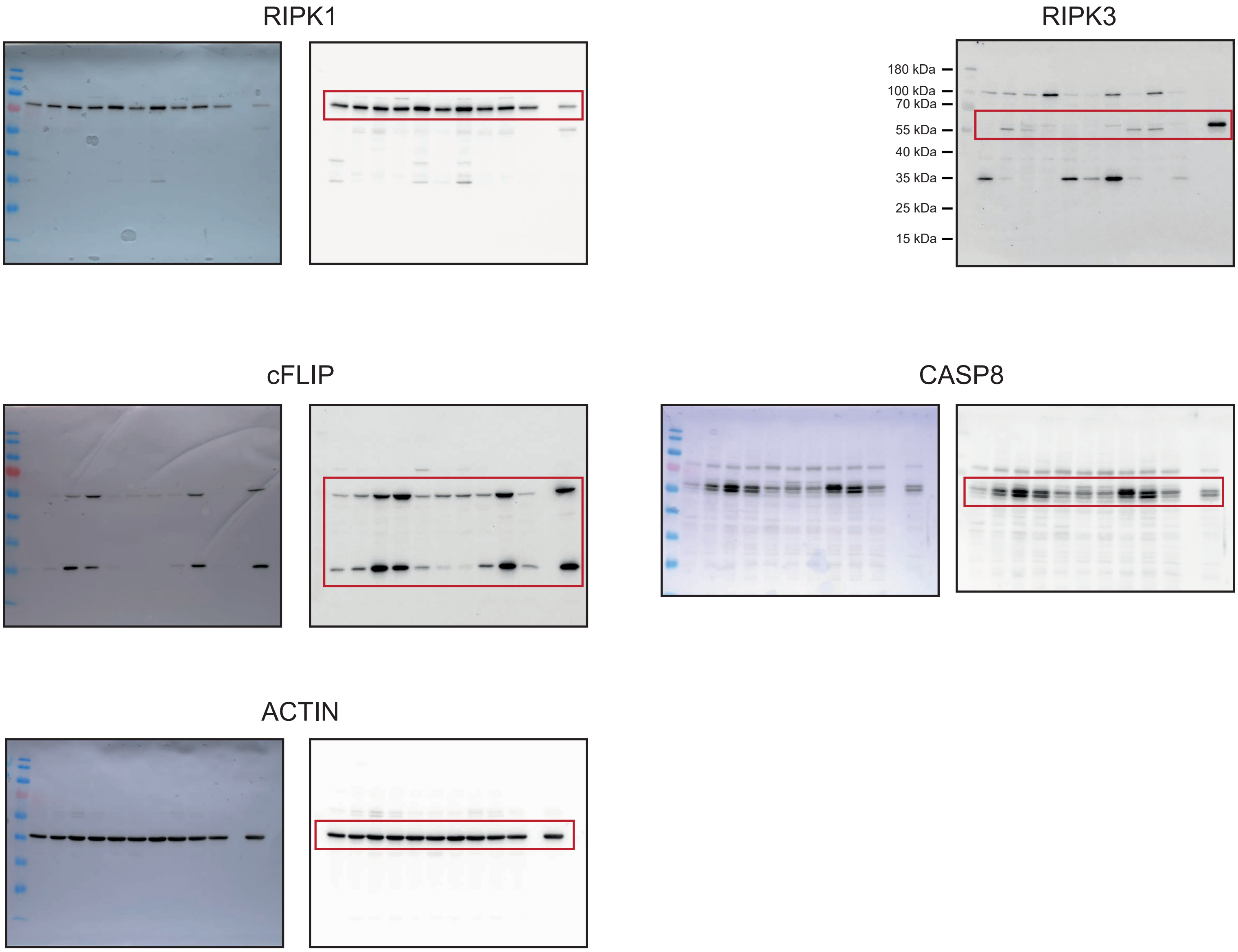

Ladder: PageRuler prestained protein ladder, 10 to 180 kDa, Thermo Fisher Scientific, Cat: 26616
Samples were loaded on different gels, beta actin was detected on one of them as sample processign control.

Figure 2H

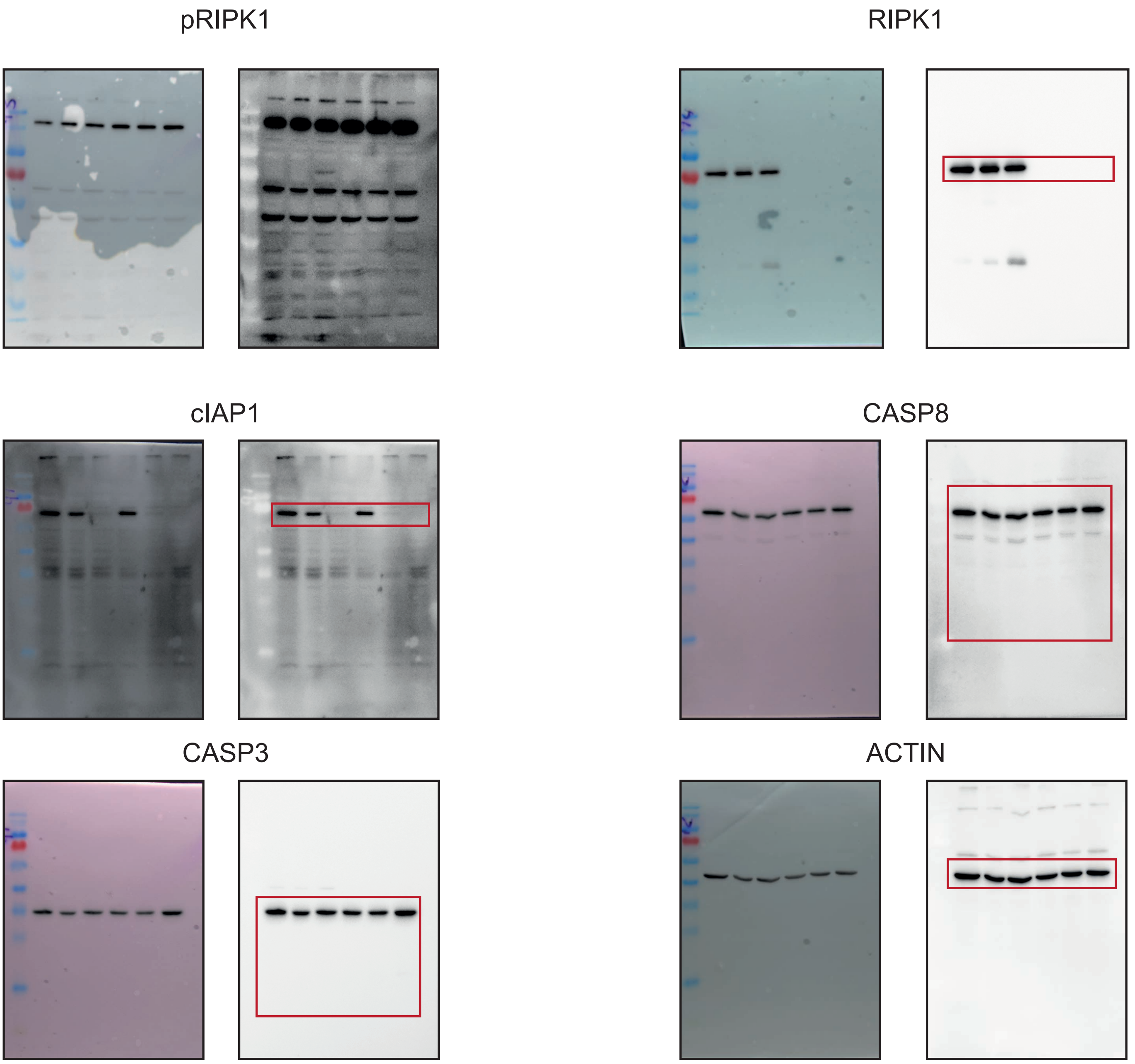

Ladder: PageRuler prestained protein ladder, 10 to 180 kDa, Thermo Fisher Scientific, Cat: 26616
Samples were loaded on different gels, beta actin was detected on one of them as sample processign control.

Figure 3D

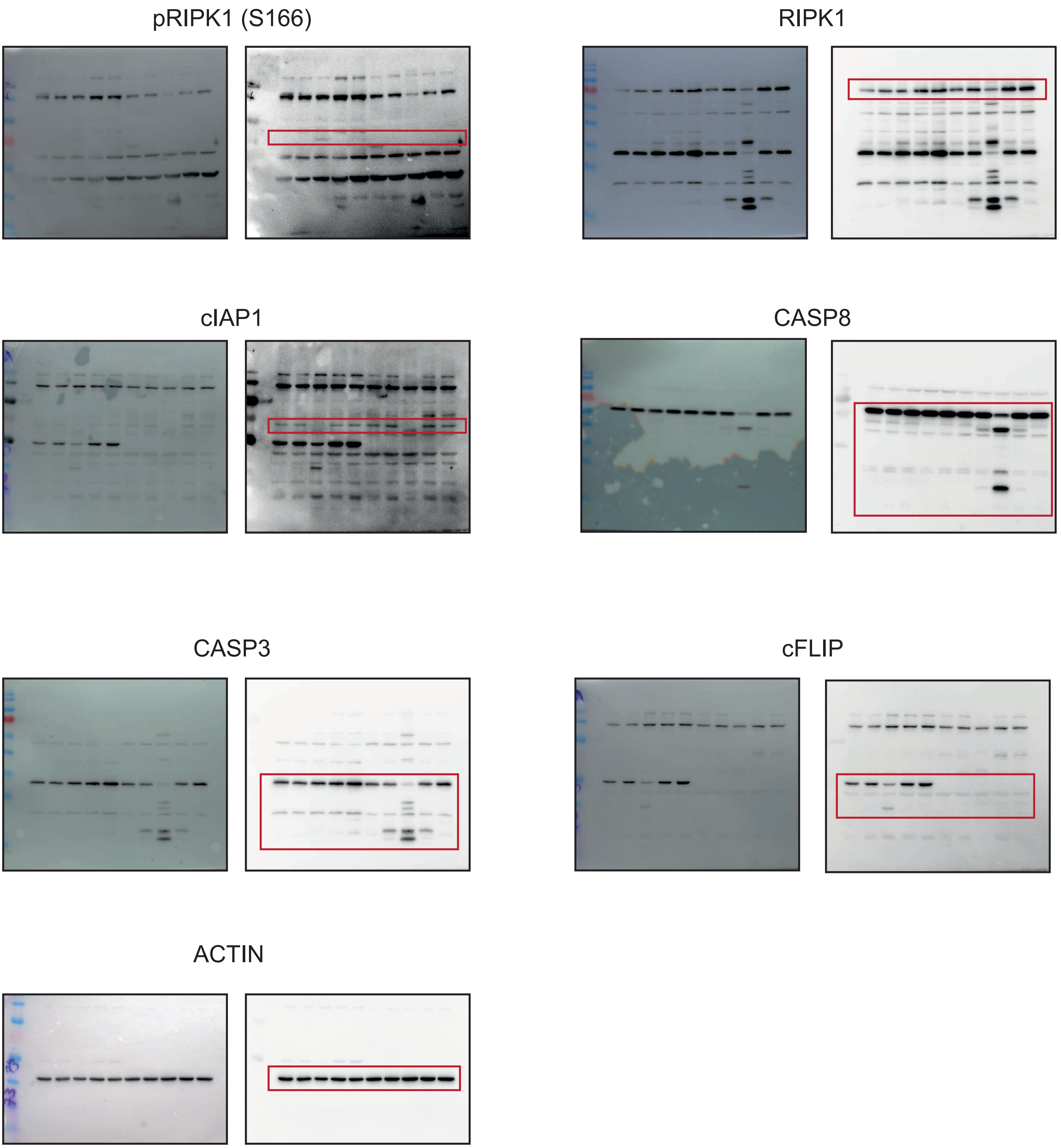

Ladder: PageRuler prestained protein ladder, 10 to 180 kDa, Thermo Fisher Scientific, Cat: 26616
Samples were loaded on different gels, beta actin was detected on one of them as sample processign control.

Figure 3E

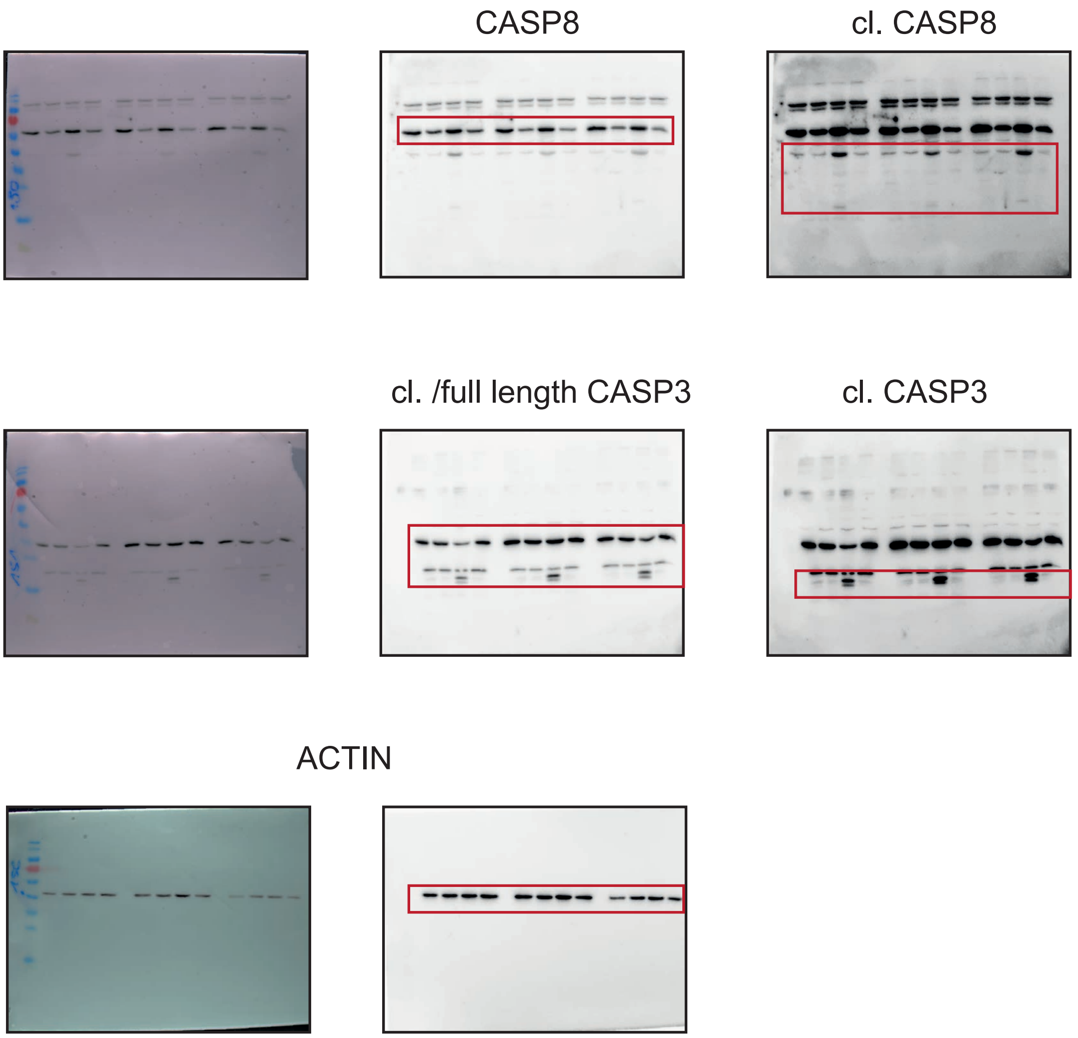

Ladder: PageRuler prestained protein ladder, 10 to 180 kDa, Thermo Fisher Scientific, Cat: 26616
Samples were loaded on different gels, beta actin was detected on one of them as sample processign control.

Figure 4A - BLM

CASP8

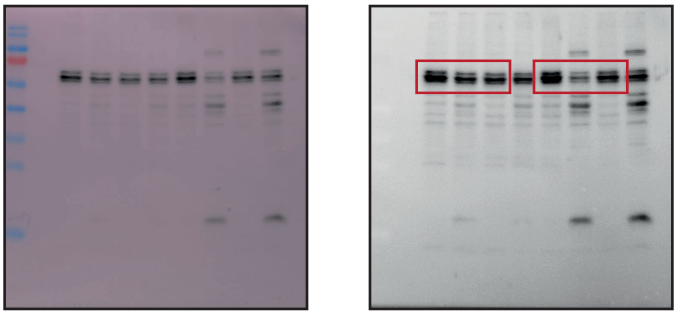

cl. CASP8

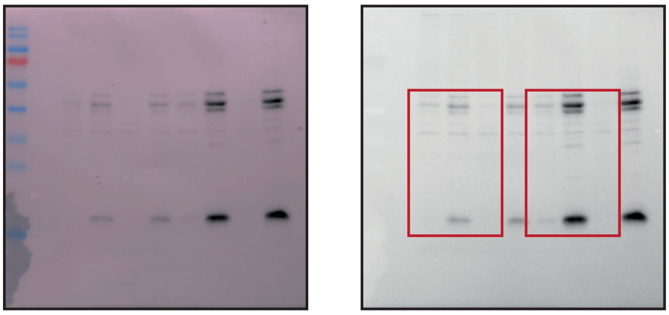

cl./full length CASP3

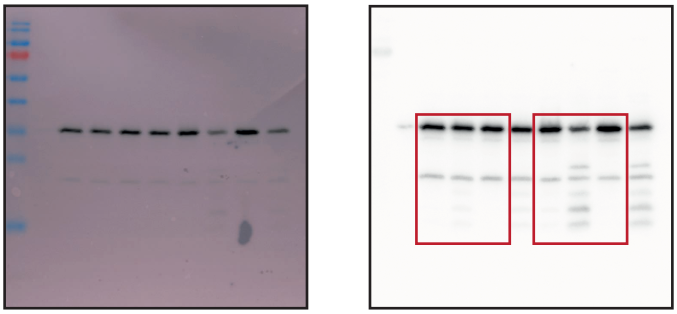

pRIPK3

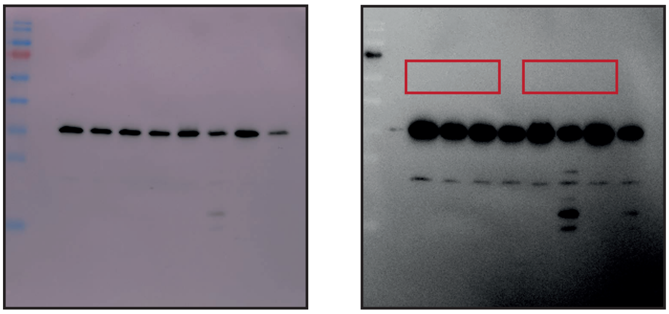

RIPK3

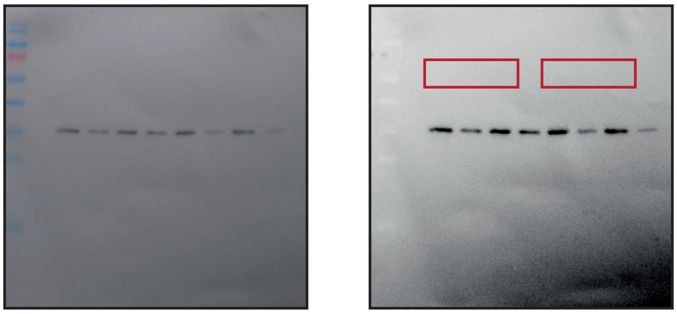

cFLIP

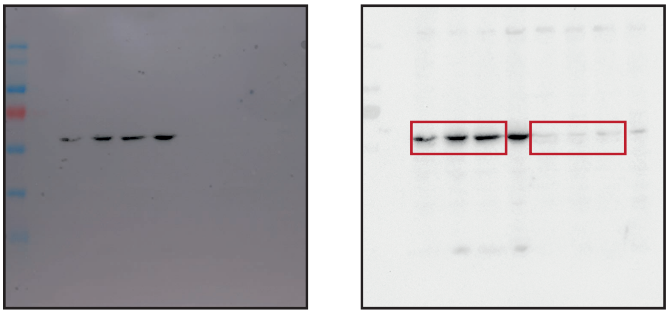

ACTIN

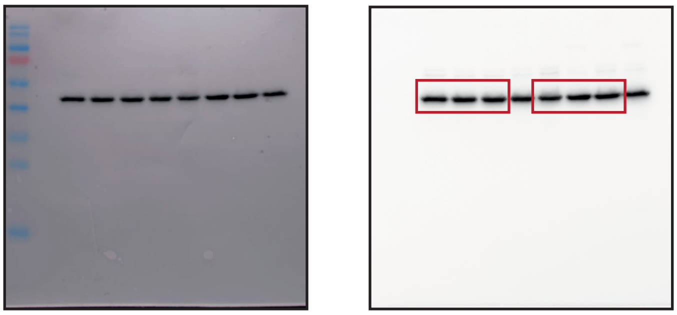

Ladder: PageRuler prestained protein ladder, 10 to 180 kDa, Thermo Fisher Scientific, Cat: 26616
Samples were loaded on different gels, beta actin was detected on one of them as sample processing control.

Figure 4A - SK-MEL-28

CASP8

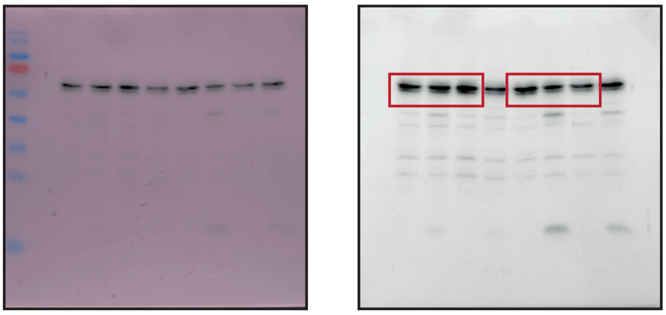

cl. CASP8

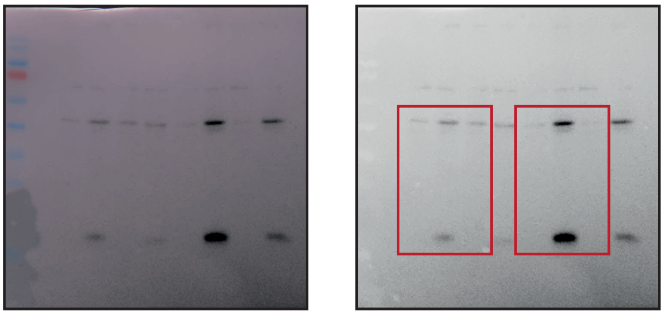

cl./full length CASP3

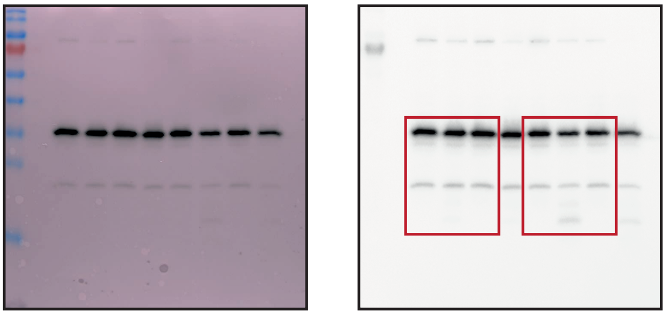

pRIPK3

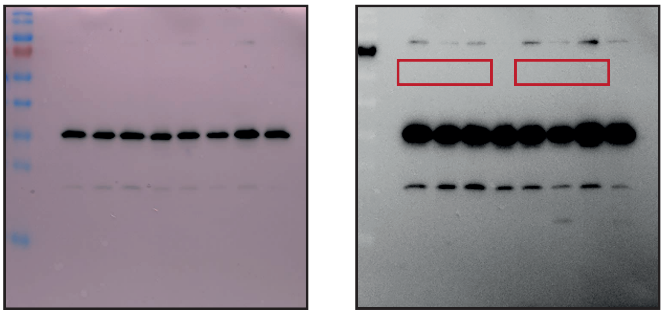

RIPK3

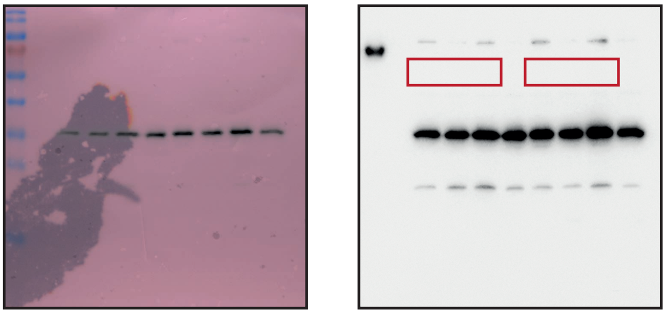

cFLIP

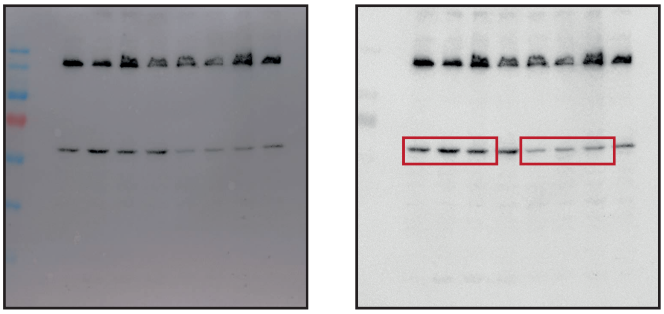

ACTIN

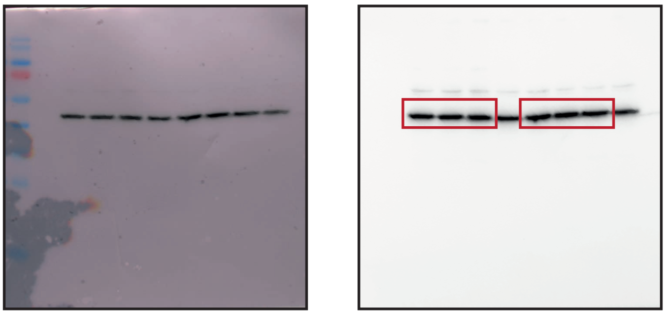

Ladder: PageRuler prestained protein ladder, 10 to 180 kDa, Thermo Fisher Scientific, Cat: 26616
Samples were loaded on different gels, beta actin was detected on one of them as sample processign control.

Figure 4A - MaMel86a

CASP8

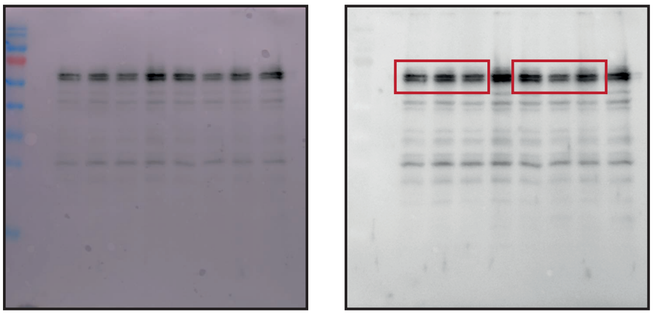

cl. CASP8

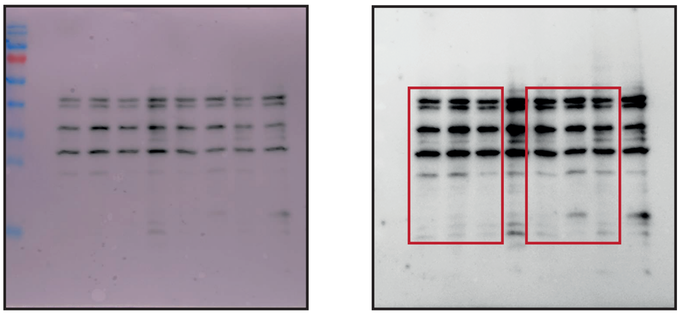

cl./full length CASP3

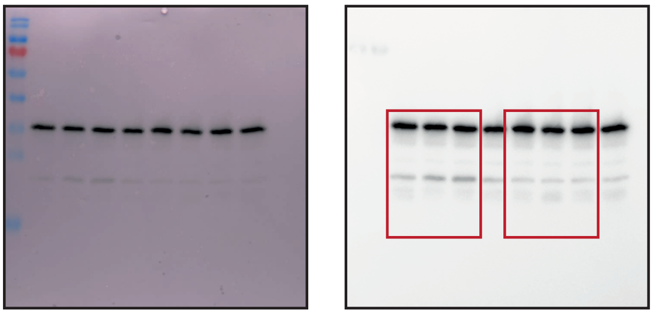

pRIPK3

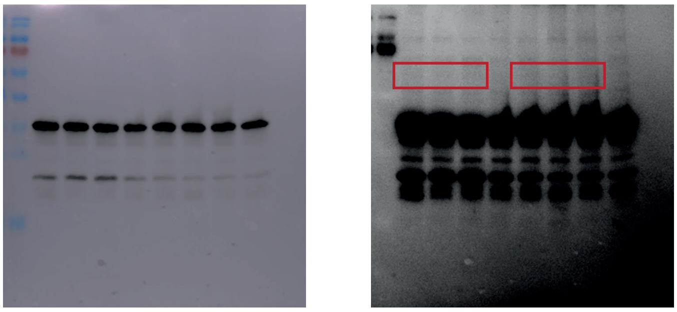

RIPK3

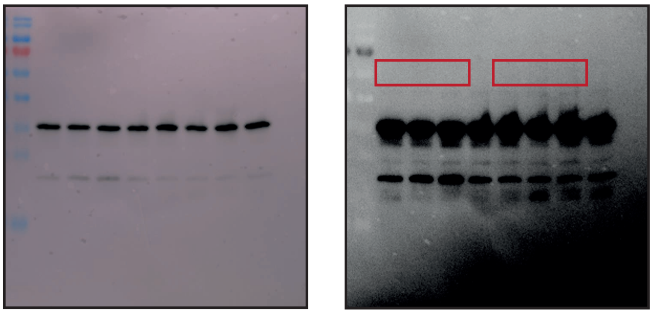

cFLIP

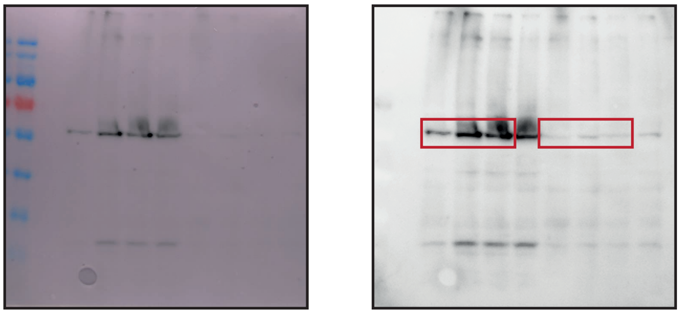

ACTIN

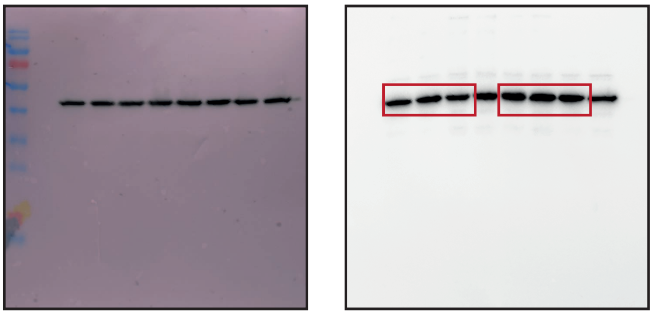

Ladder: PageRuler prestained protein ladder, 10 to 180 kDa, Thermo Fisher Scientific, Cat: 26616
Samples were loaded on different gels, beta actin was detected on one of them as sample processign control.

Figure 4A - A375

CASP8

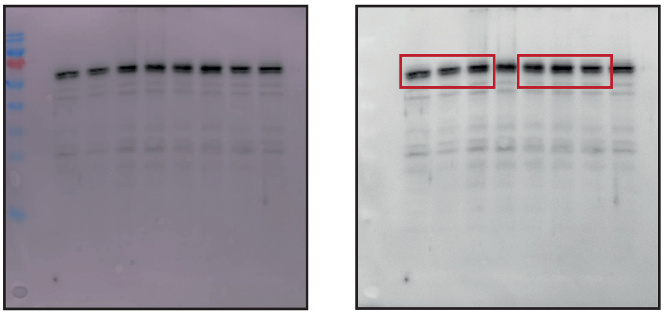

cl. CASP8

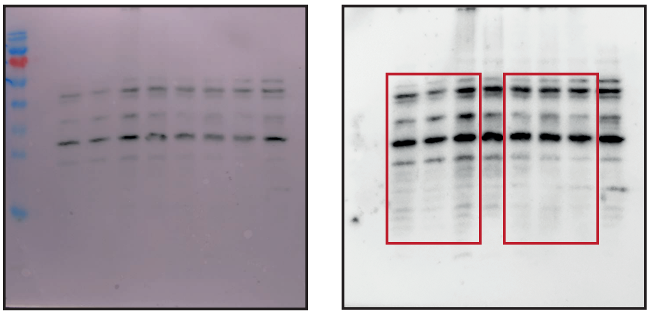

cl./full length CASP3

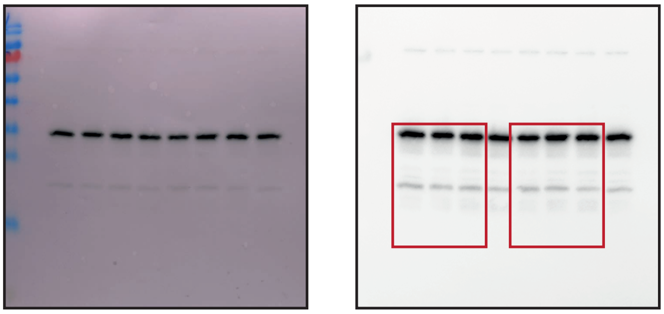

pRIPK3

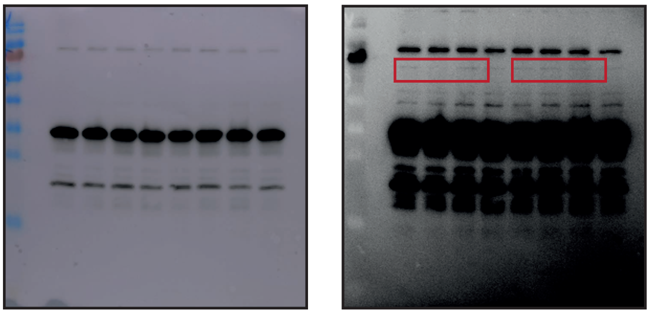

RIPK3

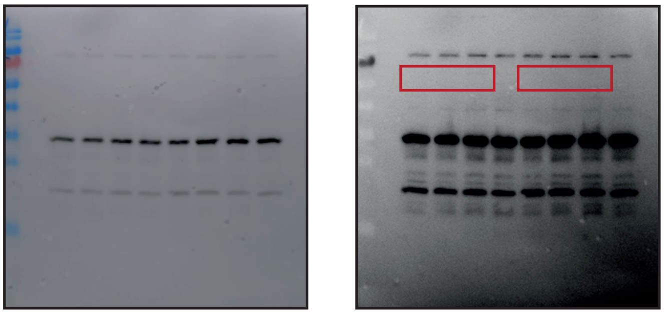

cFLIP

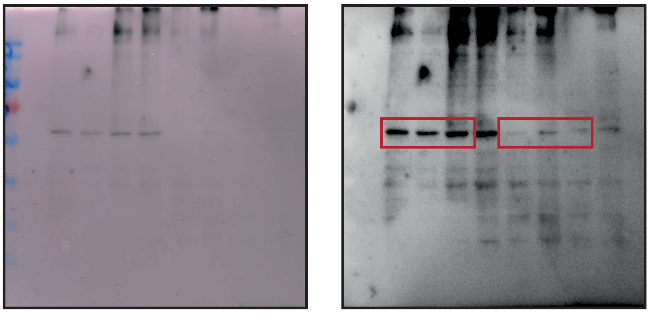

ACTIN

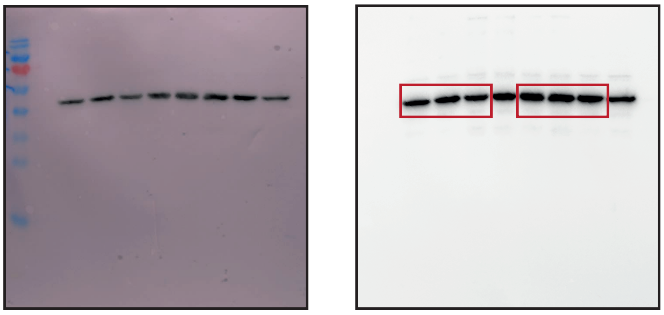

Ladder: PageRuler prestained protein ladder, 10 to 180 kDa, Thermo Fisher Scientific, Cat: 26616
Samples were loaded on different gels, beta actin was detected on one of them as sample processign control.

Supplementary Figure 1A

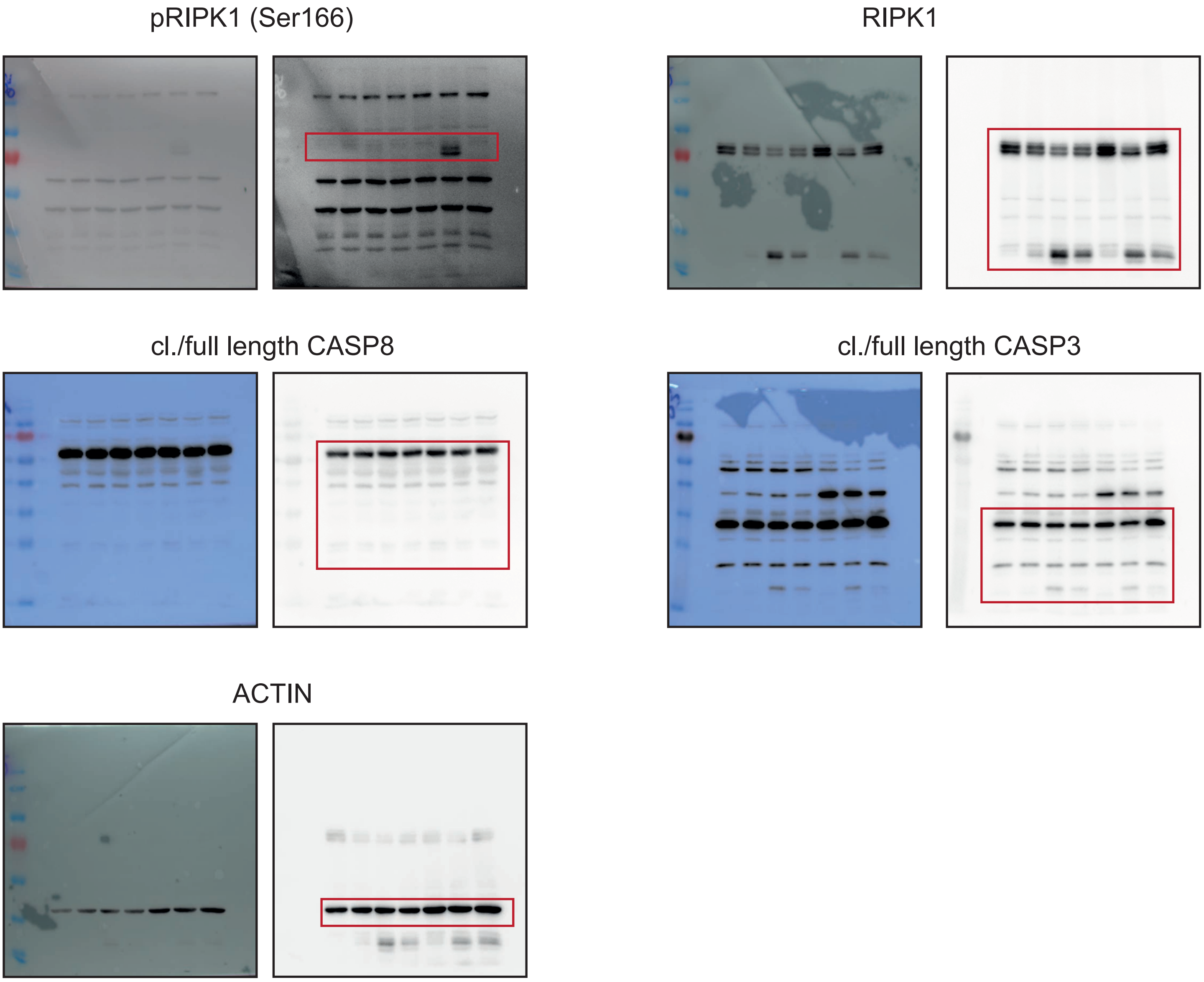

Supplementary Figure 1B

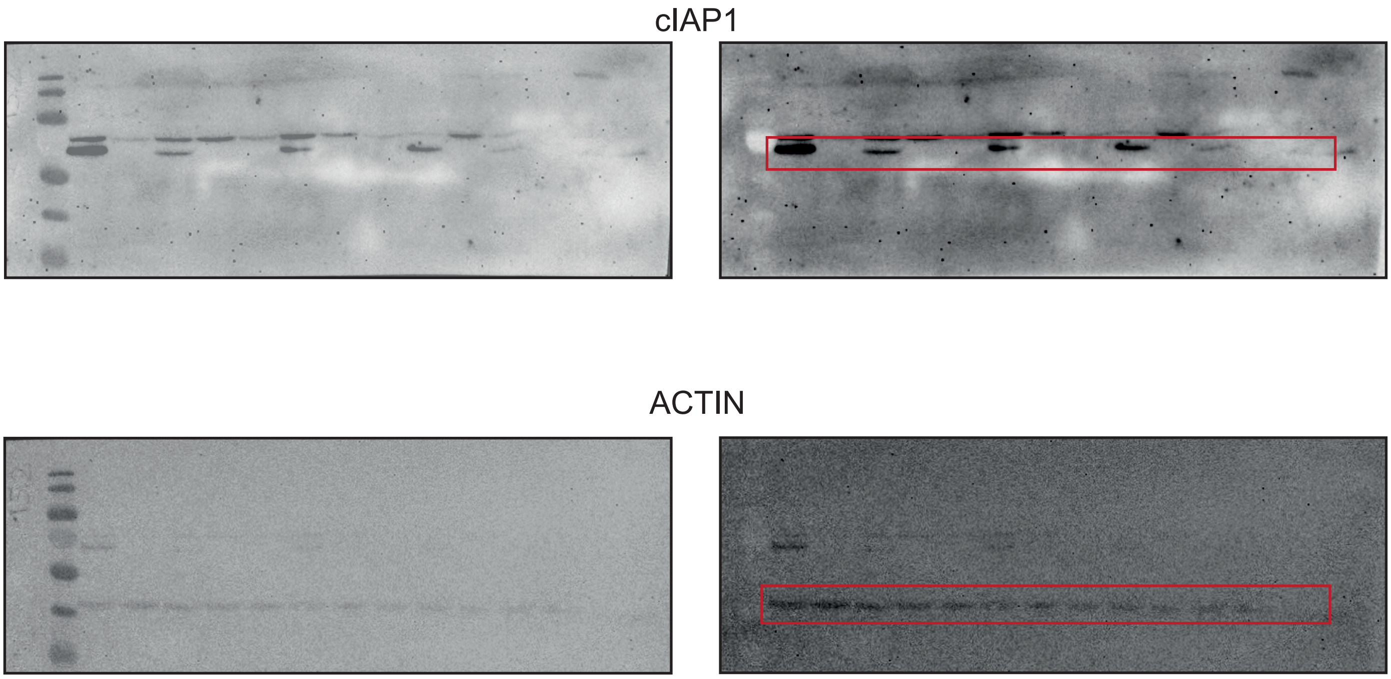

Ladder: PageRuler prestained protein ladder, 10 to 180 kDa, Thermo Fisher Scientific, Cat: 26616
S1A: Samples were loaded on different gels, beta actin was detected on one of them as sample processign control.

Supplementary Figure 2A

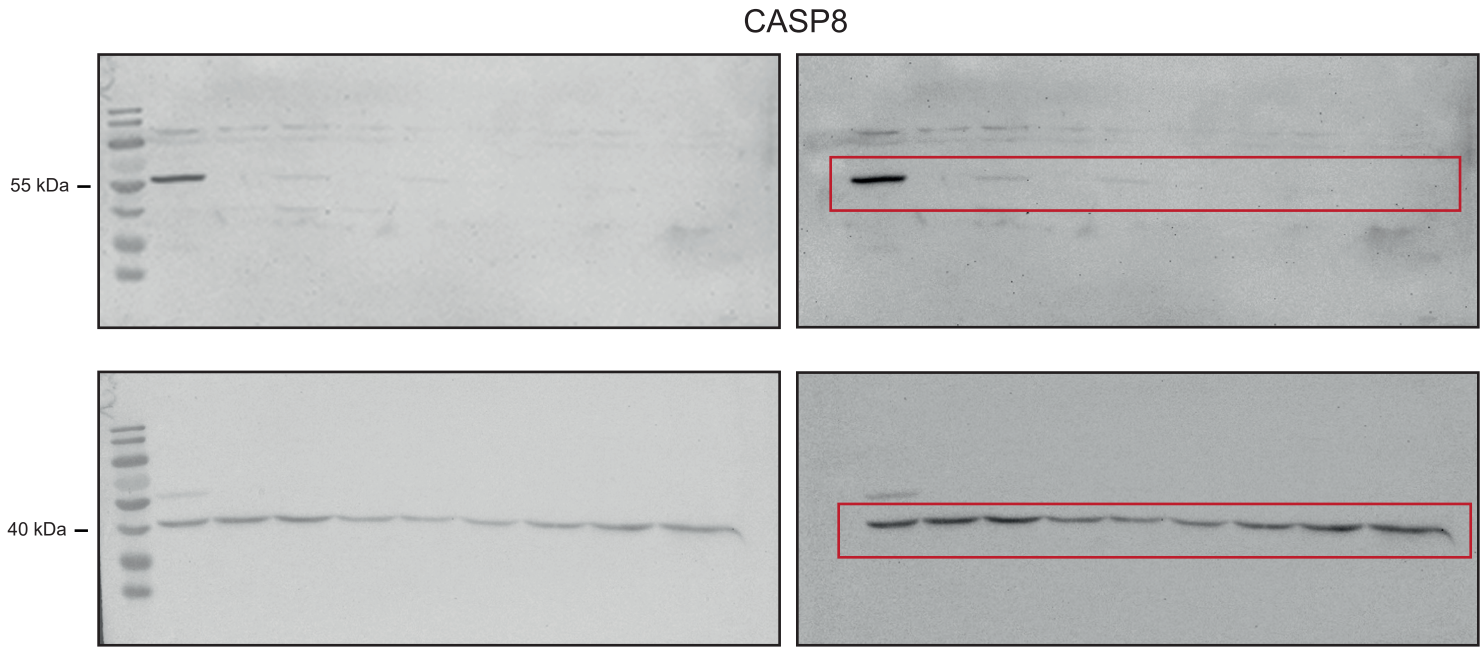

Supplementary Figure 2B

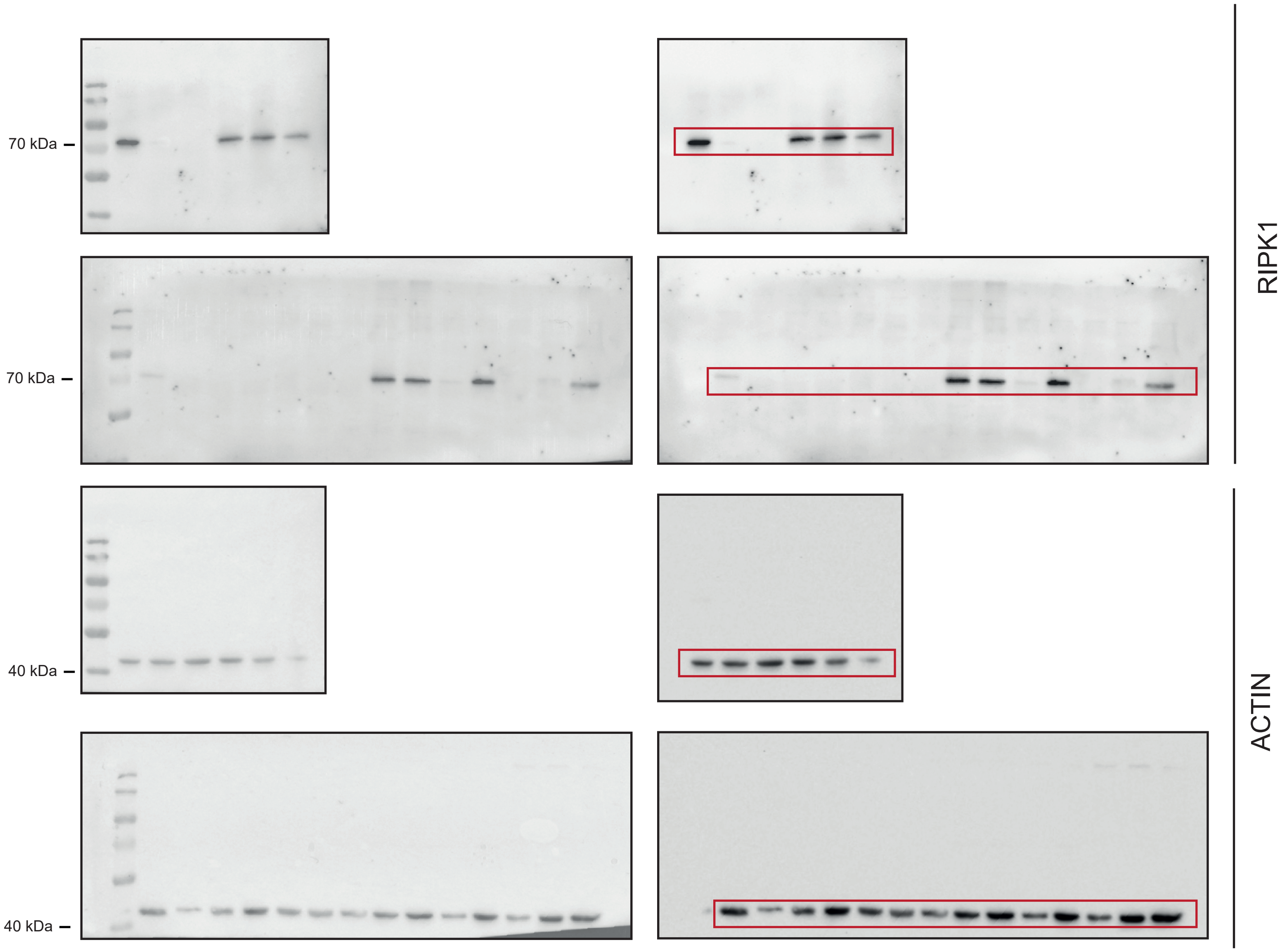

Ladder: PageRuler prestained protein ladder, 10 to 180 kDa, Thermo Fisher Scientific, Cat: 26616

Supplementary Figure 3A

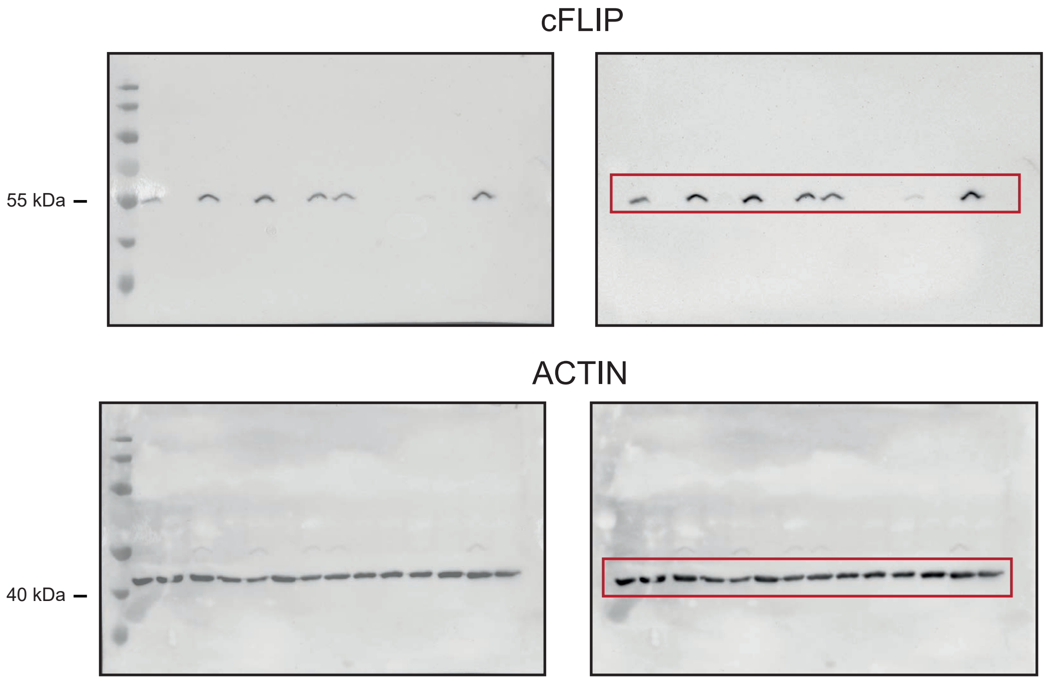

Supplementary Figure 3C

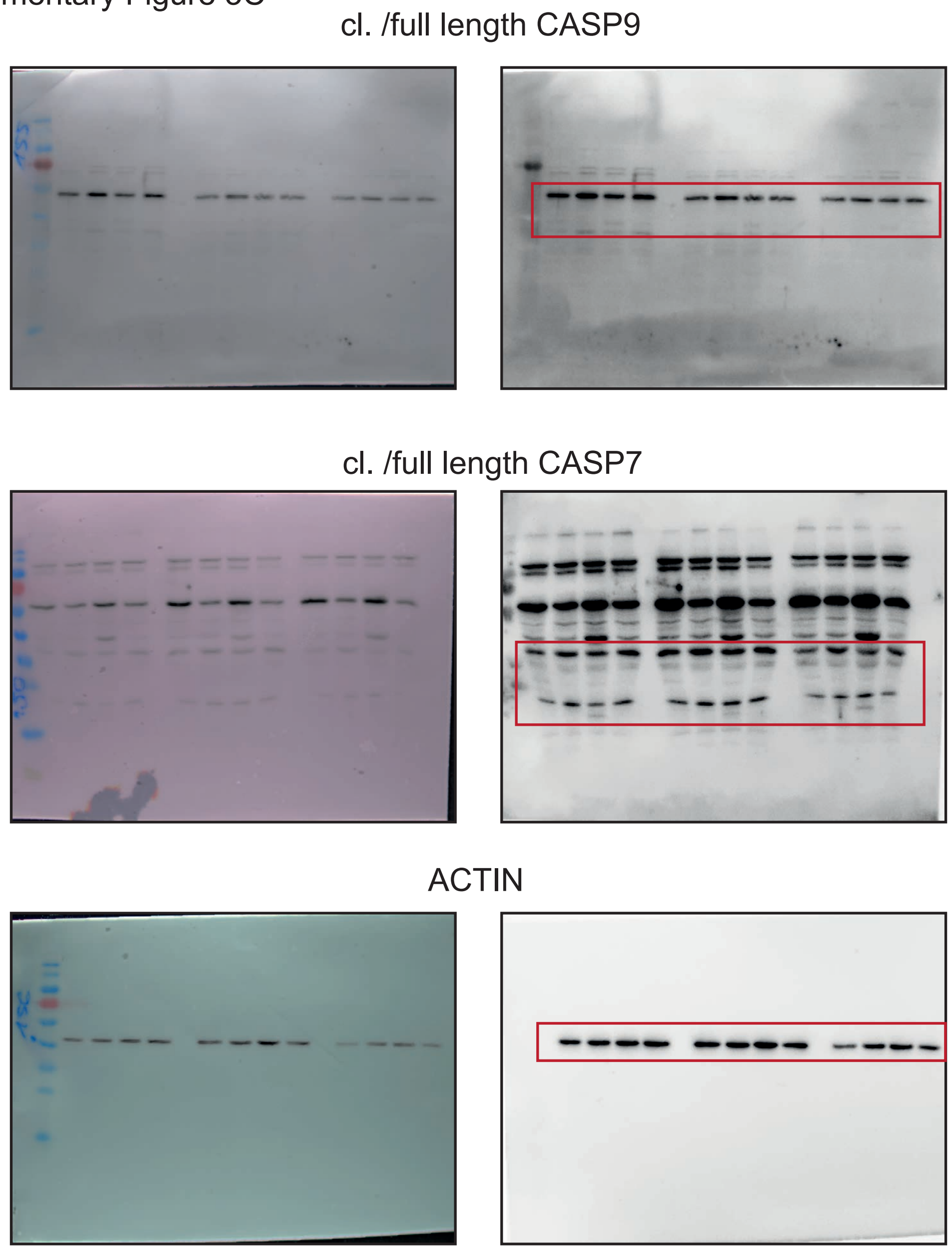

Ladder: PageRuler prestained protein ladder, 10 to 180 kDa, Thermo Fisher Scientific, Cat: 26616
S2B: Samples were loaded on different gels, beta actin was detected on one of them as sample processign control.

Supplementary Figure 4A - BLM

cFLIP

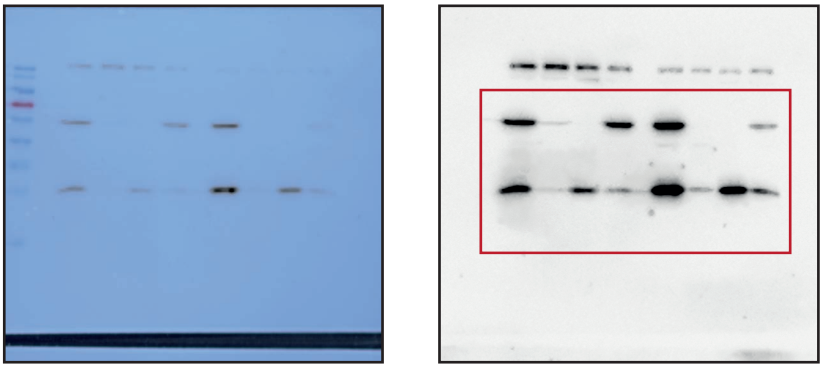

cl. /full length CASP8

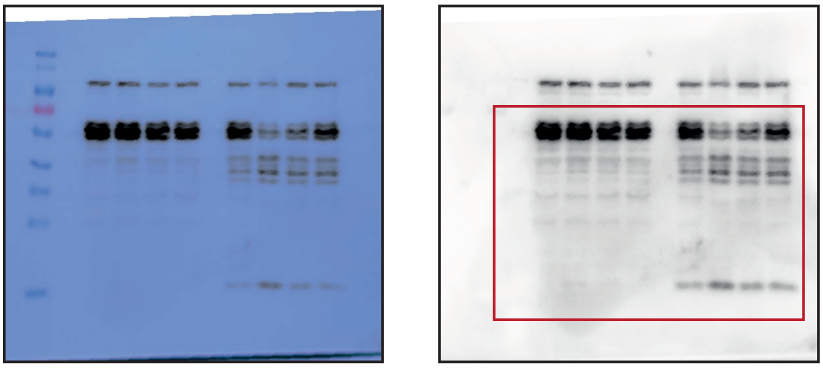

CASP3 cl. CASP3

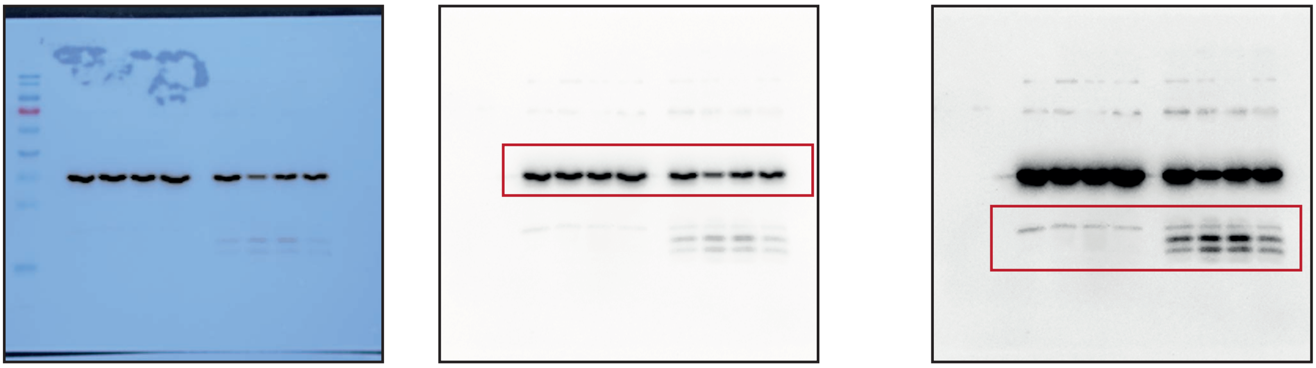

ACTIN

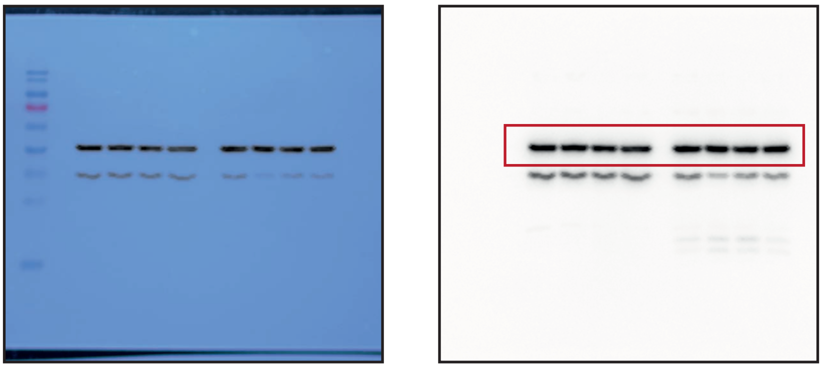

Ladder: PageRuler prestained protein ladder, 10 to 180 kDa, Thermo Fisher Scientific, Cat: 26616
Samples were loaded on different gels, beta actin was detected on one of them as sample processign control.

Supplementary Figure 4A - SK-Mel-28

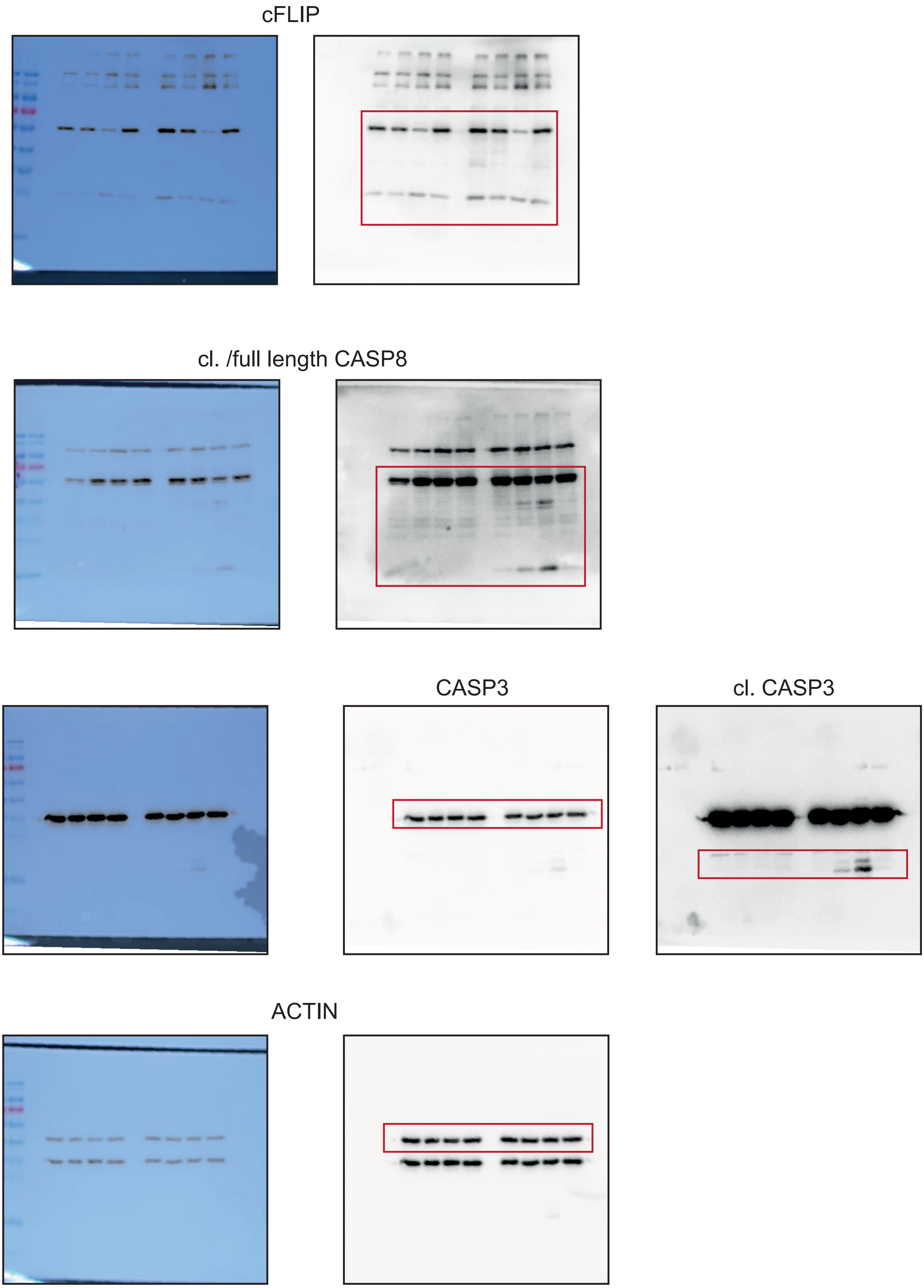

Ladder: PageRuler prestained protein ladder, 10 to 180 kDa, Thermo Fisher Scientific, Cat: 26616
Samples were loaded on different gels, beta actin was detected on one of them as sample processign control.

Supplementary Figure 4A - MaMel86a

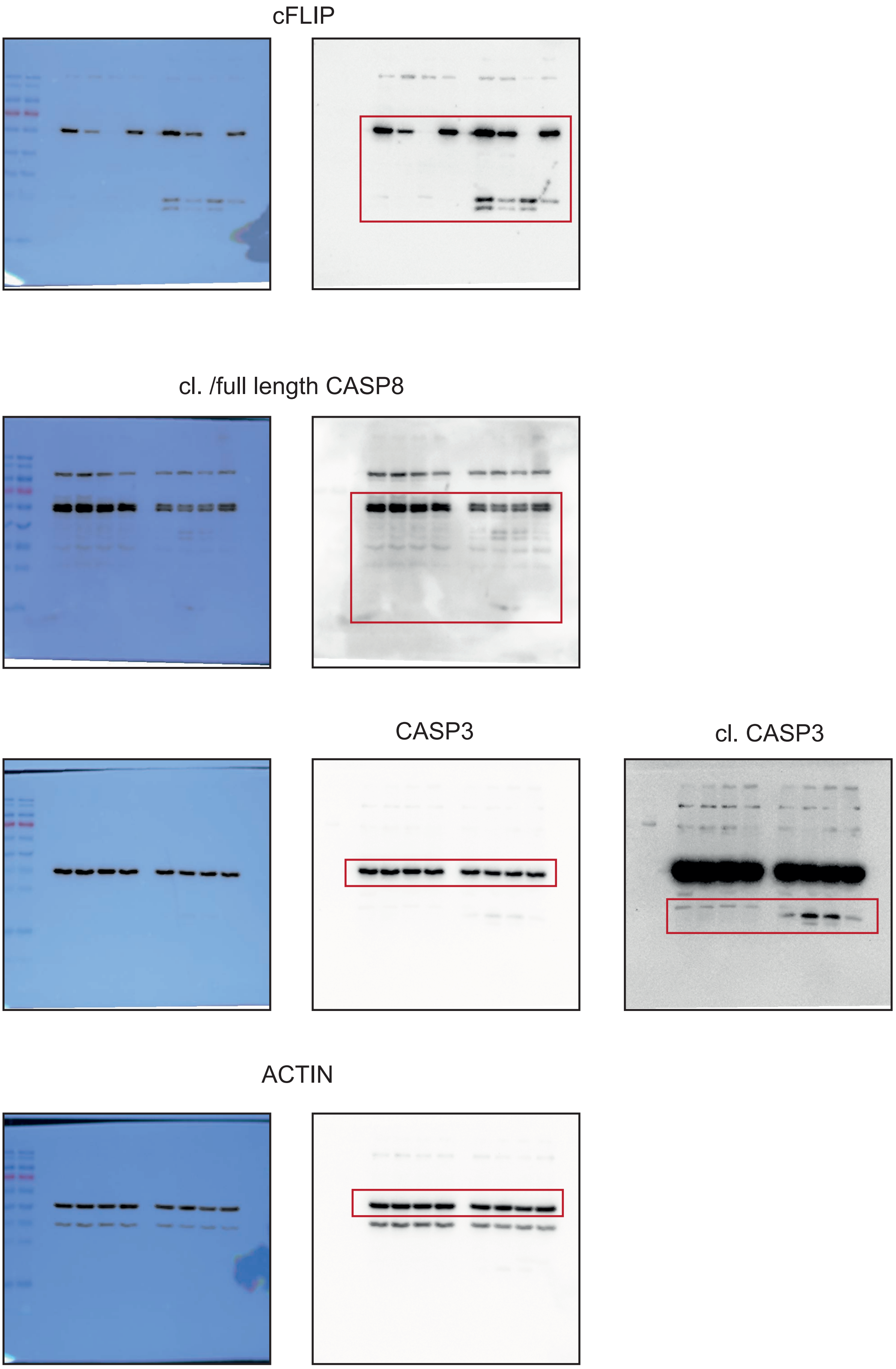

Ladder: PageRuler prestained protein ladder, 10 to 180 kDa, Thermo Fisher Scientific, Cat: 26616
Samples were loaded on different gels, beta actin was detected on one of them as sample processign control.

Supplementary Figure 4A - A375

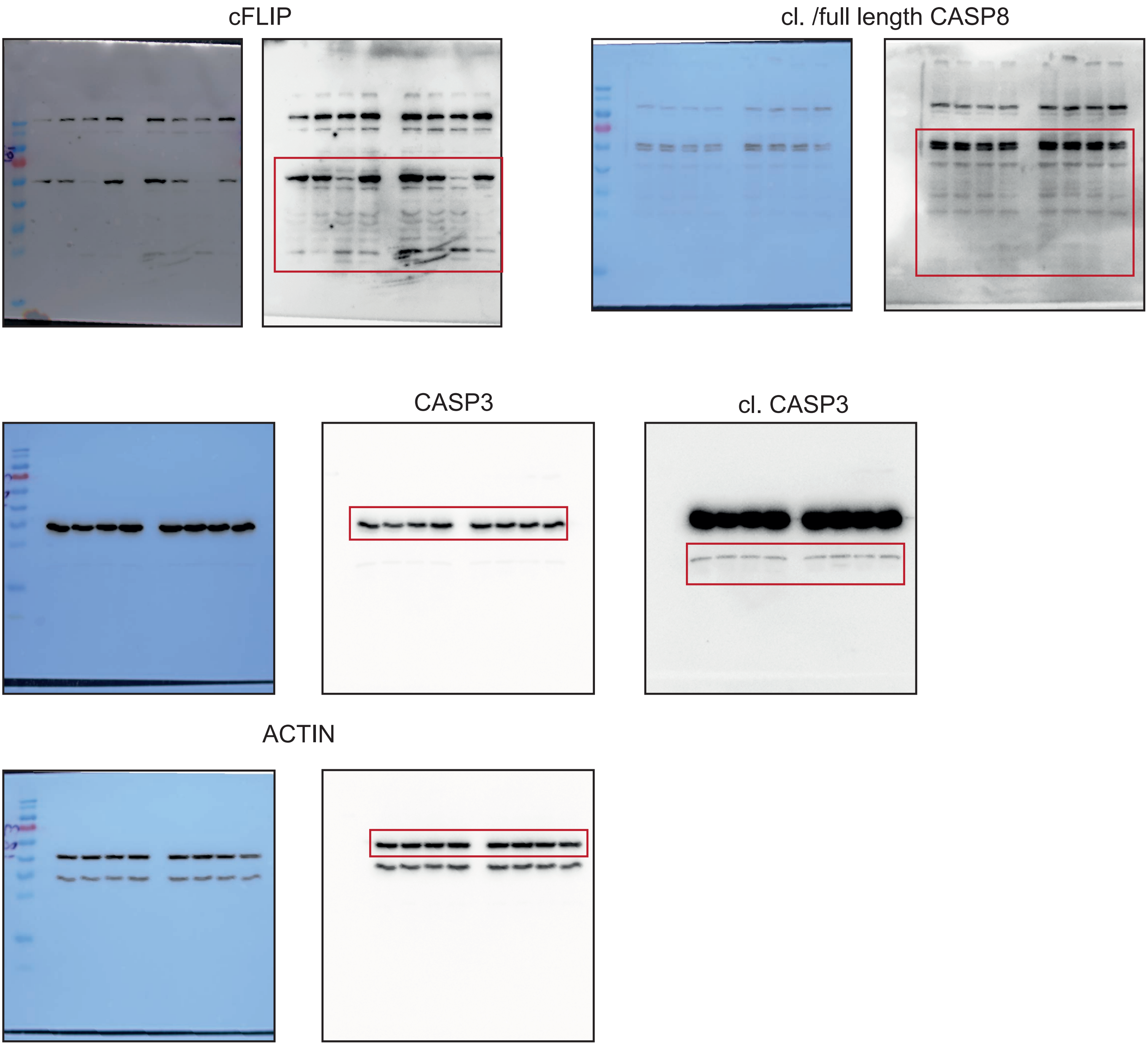

Ladder: PageRuler prestained protein ladder, 10 to 180 kDa, Thermo Fisher Scientific, Cat: 26616
Samples were loaded on different gels, beta actin was detected on one of them as sample processign control.
